# Advanced Human Immune Cell‐Organoid Co‐Cultures for Functional Testing of Cancer Nanovaccines

**DOI:** 10.1002/advs.202515199

**Published:** 2025-12-23

**Authors:** Nathalia Ferreira, David Agorku, Andre Rosa, Julia Roosz, Lena Christ, Nicole Anderle, Ajinkya Kulkarni, Abir Hussein, Sana S. Sayedipour, Omar F. Luna, Tobias Legler, Philipp Ströbel, Fernando Albericio, Luis Cruz, Phillipp Beckhove, Peter Loskill, Frauke Alves, M. Andrea Markus, Fernanda Ramos‐Gomes

**Affiliations:** ^1^ Translational Molecular Imaging Max‐Planck‐Institute For Multidisciplinary Sciences Göttingen Germany; ^2^ Miltenyi Biotec B.V. & Co. KG Bergisch Gladbach Germany; ^3^ NMI Natural and Medical Sciences Institute at the University of Tübingen Reutlingen Germany; ^4^ Division of Interventional Immunology Leibniz Institute for Immunotherapy Regensburg Germany; ^5^ Department of Radiology Leiden University Medical Center Leiden Netherlands; ^6^ Department of Organic Chemistry, University of Barcelona, and CIBER‐BBN, Networking Centre on Bioengineering Biomaterials, and Nanomedicine Barcelona Spain; ^7^ Department of Transfusion Medicine University Medical Center Göttingen Göttingen Germany; ^8^ Institute of Pathology University Medical Center Göttingen Göttingen Germany; ^9^ Department of Internal Medicine III University Hospital Regensburg Regensburg Germany; ^10^ Department of Microphysiological Systems Institute of Biomedical Engineering Faculty of Medicine Eberhard Karls University‐Tübingen Tübingen Germany; ^11^ 3R Center Tübingen for In Vitro Models and Alternatives to Animal Testing Tübingen Germany; ^12^ Clinic of Hematology and Medical Oncology and Department of Diagnostic and Interventional Radiology University Medical Center Göttingen Göttingen Germany

**Keywords:** cancer vaccine, immunotherapy, mesothelin, organoids, PDAC

## Abstract

Pancreatic ductal adenocarcinoma (PDAC) remains a major clinical challenge due to late detection and limited treatment responsiveness. To better evaluate complex immunotherapies in a human‐relevant setting, we developed an integrated organoid–immune co‐culture pipeline using PDAC patient‐derived organoids (PDOs) and matched HLA immune cells. As a proof of concept, we assessed an MSLN‐targeted nanovaccine (Mesovac), alone and in combination with FOLFIRINOX chemotherapy and Atezolizumab. We evaluated Mesovac across a multi‐stage pipeline, including T‐cell stimulation, ex vivo expansion, and PDO‐immune co‐cultures, to assess immune activation, specificity, and synergy with combinatorial treatments. MSLN‐stimulated T‐cells, derived from PDAC patients, showed increased IFN‐γ production and selective infiltration into MSLN‐expressing PDOs. Artificial antigen‐presenting cells (aAPCs) boosted the expansion of reactive T‐cells, enhancing antitumor responses. Notably, combining Mesovac with FOLFIRINOX and Atezolizumab maintained PD‐L1+ T‐cell levels and reduced cancer stem cells and aggressive PDAC subsets. Using this advanced in vitro workflow, we highlight that this platform, using human organoid–immune cell co‐cultures, enables the evaluation of complex processes related to nanovaccine strategies that would not be possible in vivo.

## Graphical abstract

Pancreatic ductal adenocarcinoma remains difficult to treat. We establish an organoid–immune co‐culture using patient‐derived organoids and matched T‐cells to assess cancer vaccines. A mesothelin‐targeted nanovaccine activates antigen‐specific T‐cells, increases IFN‐γ, and targets MSLN+ organoids. Combined with FOLFIRINOX and Atezolizumab, it reduces stemness and maintains immune activity, enabling personalized ex vivo vaccine evaluation.

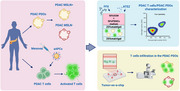

## 1 Introduction

Pancreatic Ductal Adenocarcinoma (PDAC) is the most prevalent neoplastic disease of the pancreas, accounting for more than 90% of all pancreatic malignancies [1]. PDAC, with its aggressive nature and resistance to conventional therapies, remains hard to treat. Presently, the treatment approach for PDAC involves surgical intervention for tumors that can be removed, and the use of chemotherapy regimens, such as paclitaxel together with the nucleoside analog gemcitabine, or FOLFIRINOX, a combination therapy of four drugs that includes 5‐fluorouracil (5‐FU), leucovorin, irinotecan, and oxaliplatin. This chemotherapy is applied in various scenarios for patients with advanced PDAC, including neo‐adjuvant, adjuvant, or palliative care. Unfortunately, these treatments lead to only a slight increase in survival rates [2].

Recently, immunotherapy has emerged as a promising approach to cancer treatment, providing fewer side effects and less tumor resistance than conventional therapies [3]. However, in PDAC, several mechanisms of immune escape are able to counteract the therapeutic responsiveness to immunotherapy. The PDAC immunosuppressive and immune evasive microenvironment, characterized by dense stromal elements and limited effector T‐cell infiltration, represents an obstacle that limits the effectiveness of immune checkpoint inhibitors and other immunotherapies [4]. Overall, the use of immunotherapy in PDAC could be improved with the design of rational combinations with chemotherapy [5]. The most extensively evaluated immunotherapy approach in PDAC has involved the use of vaccines, which are diverse and employ very different mechanisms. Cancer vaccines operate based on the same mechanisms as other diseases, where the key component initiates an immune reaction that results in enduring immunity against a foreign antigen. These vaccines educate the immune system to recognize tumor antigen as foreign, leading to the identification and destruction of cancer cells [6].

Here, mesothelin (MSLN), a cell surface glycoprotein highly expressed in PDAC, represents a promising target for immunotherapeutic interventions [7]. In vitro and in animal models, it was shown to have the potential for precise targeting, limiting off‐target effects in normal cells, and inhibiting or eradicating cancer cells resistant to traditional treatments [7, 8]. Ranging from monoclonal antibodies (mAb), namely Amatuximab, to Immunotoxins SS1P and LMB‐100/RG7787, 227Th‐radiolabeled antibody, antibody‐drug conjugates, vaccines to CAR‐T‐cells, MSLN, in spite of an incompletely resolved mode of action, remains an attractive target, and several clinical trials are currently ongoing [9]. As a cancer vaccine targeting MSLN in PDAC, GVAX, a GM‐CSF gene‐transfected tumor cell vaccine, has been shown to induce specific T‐cell responses against MSLN, correlating with longer survival in PDAC patients [10]. Another vaccine, CRS‐207, based on bacterial engineering to express MSLN, although promoting immune‐activation, did not yield significant tumor responses alone [11]. Combining CRS‐207 with GVAX and the chemotherapeutic drug cyclophosphamide improved survival initially but failed in a larger study involving PDAC patients [12]. While cancer vaccines have shown promise in preclinical studies, their translation into effective treatments for PDAC remains challenging, partly due to PD‐L1 upregulation hindering the immune response [13].

A novel approach for improvement of antigen immunogenicity is the formulation of vaccines in a nanoparticulate delivery system, as it offers several distinct advantages for cancer immunotherapy. Nanoparticles (NPs), due to their small size, enable precise delivery of tumor antigens to immune cells, enhancing antigen presentation and triggering a potent immune response against cancer cells [14]. NPs have been engineered to target specific cancer cells, minimizing damage to healthy tissues and reducing side effects. Additionally, NPs have a prolonged circulation time, which ensures a sustained immune response, optimizing the chances of effectively targeting and eliminating cancer cells [15]. Furthermore, the customizable nature of NP vaccines allows tailoring of antigens to the patient`s tumor profile, ensuring a personalized treatment approach [16].

A further slowdown for a prompt translation of novel immunotherapies into clinical application is the limited preclinical testing models available to date. Traditional animal models, such as mice, have limitations that hinder the evaluation of cancer vaccines. These models frequently fail to replicate the distinct tumor microenvironment, immune evasion mechanisms, and genetic heterogeneity characteristic of PDAC [17]. To bridge the translational gap, patient‐derived organoids (PDOs) as 3D cell cultures that retain the ability to self‐renew and self‐organize into mini‐organ‐like structures have emerged as invaluable tools for studying and testing potential treatments for PDAC, characterized by a remarkable inter‐ and intra‐tumoral heterogeneity and complexity. Several studies on transcriptional profiling of patient PDAC specimens have indicated the existence of multiple tumor subtypes, each with a varied response to different treatments, making a one‐size‐fits‐all approach rather unlikely [18]. However, a major limitation of patient‐derived organoid (PDO) models is their lack of immune cell components, which restricts their capacity to effectively evaluate immunotherapies [19]. To address this, we introduced a co‐culture strategy based on PDAC PDOs and stimulated T‐cells [20, 21], providing a comprehensive platform to assess the efficacy of a nanoparticle‐based mesothelin‐targeted nanovaccine, either alone or in combination with chemotherapy (FOLFIRINOX) and immunotherapy (Atezolizumab). This approach, integrating immune cell response profiling, efficacy, and specificity of the nanovaccine formulation, represents a step forward in the in vitro evaluation of cancer vaccines by utilizing PDAC organoid/immune cell co‐cultures as an optimal model for testing personalized treatment regimens tailored to individual patient profiles.

## 2 Materials and Methods

### 2.1 Establishment of PDAC Patient‐Derived Organoids

PDOs were generated from human PDAC specimens following standard organoid cell culture methods established as described in [22]. PDAC tissue was extracted from the tumor bulk following surgical resection and confirmation of a PDAC diagnosis. All research involving human PDAC material obtained from patients participating in the Molecular Pancreas Program (MolPAC) at the University Medical Center Goettingen, which includes the generation and utilization of patient‐derived organoids, has received approval from local regulatory authorities (approval references: 11/5/17, 22/8/21Ü, and 2/4/19). For organoid culturing, a detailed protocol for the preparation of organoid medium can be found in the supplementary data.

### 2.2 Evaluation of MSLN Expression in PDAC by Immunohistochemistry (IHC)

#### 2.2.1 Paraffin Embedding of Patient‐Derived Organoids

Following an established protocol [23], human PDAC PDOs were prepared for paraffin embedding. In brief, the Digestion Cell Recovery Medium (Corning) was added directly into the organoid domes and incubated for 30 min at 37°C to facilitate Matrigel removal from the organoids. Subsequently, a 200 µL tip was used to excise the organoids, which were transferred into a round‐bottom cryotube vial and centrifuged at 200xg for 30 s. After removal of the medium, the organoids were fixed by incubating them in 4% paraformaldehyde (PFA) for 40 min followed by centrifugation at 200xg for 30 s and embedding in 0.5% melted agarose (BioFroxx). The tube was inverted after embedding, and the agarose‐embedded PDOs were again spun down at 200xg for 30 s. To facilitate agarose solidification, the inverted vial was incubated at 4°C for 10 min. Subsequently, the solidified agarose organoid block was retrieved using a pair of fine‐tipped tweezers and embedded in a cassette before being immersed in 4% PFA. For paraffin embedding, the agarose organoid block was rinsed briefly in phoshate buffered saline (PBS) and subsequently underwent a series of ethanol immersions: three times in 70% ethanol, two times in 90% ethanol, and three times in 100% ethanol, each lasting 30 min. Following the dehydration protocol, the block was immersed three times in xylol for 20 min each before embedding in paraffin at 58°C (Thermo Scientific, HistoStar) for 30 min two times. For the paraffin block solidification, paraffin‐embedded PDOs were placed on a cooling plate. Sections of the paraffin‐embedded organoids, each measuring 4 µm in thickness, were prepared using a microtome (Leica RM2255). Subsequently, adhesive slides (Epredia) were placed in a water bath, and the organoid sections were mounted on slides and dried at 37°C for 30 min for subsequent IHC staining.

#### 2.2.2 IHC to Assess Mesothelin Expression on PDAC Tissues and Organoids

Paraffin sections from human PDAC PDOs were subjected to IHC staining to assess MSLN expression. Initially, paraffin removal was achieved through incubation of slides at 60°C for 30 min, followed by two washes in xylene for 7 min each and a single wash in propanol for 5 min. Subsequently, the sections underwent a rehydration process through a series of ethanol immersions: 98% ethanol, 75% ethanol, 60% ethanol, and water for 5 min each. For antigen retrieval, the sections were exposed to a heated commercial antigen unmasking solution (DAKO) within a steamer (Braun 3216) at 97°C for 20 min, followed by cooling to room temperature (RT) for an additional 20 min. The slides were then washed twice for 5 min each with a solution containing 0.025% Triton X‐100 in Tris‐Buffered Saline (TBS). Subsequent blocking was achieved using the SEABLOCK reagent (ThermoScientific). Following this, the primary rabbit polyclonal antibody to human MSLN (LsBio, 1:150, MB‐G10) diluted in commercial diluent (DAKO) was applied and incubated overnight at 4°C. After two 5 min washes with a solution of 0.025% Triton X‐100 in TBS, a mixture of 0.3% hydrogen peroxide (Sigma) in TBS was added to block endogenous peroxidase activity for 15 min. After two 5 min washes with 0.025% Triton X‐100 in TBS, the slides were incubated with an anti‐rabbit secondary antibody (Histofine) for 1 h at RT. After a 5 min rinse in running tap water, an AEC substrate (BD Pharmingen) was applied to detect peroxidase enzyme activity. Upon chromogen development, the slides were washed for 5 min under running tap water and counterstained by immersing them in hematoxylin for 5 s. Finally, the slides were washed under running water for 5 min and mounted using the mounting medium Aquapolymount (Polysciences).

### 2.3 qPCR Analysis of MSLN Expression in PDAC Organoids

Fully matured PDOs were harvested and subjected to mechanical dissociation using a 200 µL tip affixed to a 10 mL serological pipette. A single‐cell suspension was subsequently obtained by incubating the organoids for 3 min with Trypsin/EDTA (0.25%/0.02% (w/v) in PBS) solution at 37°C. Following this, a cell density of 1 × 10^4 cells was mixed with 15 µL of Matrigel and plated in 48‐well plates using HOGM, as outlined in the protocol available at http://tuvesonlab.labsites.cshl.edu/wpcontent/uploads/sites/49/2018/06/20170523_OrganoidProtocols.pdf, with Y‐2732 supplementation. After 3 days, matured organoids were collected for mRNA extraction. Pellets were washed with cold PBS and centrifuged to remove traces of Matrigel. Total RNA was isolated from pelleted cells using the RNeasy Micro kit (Qiagen), and 90 ng of RNA were transcribed to cDNA using the QuantiTect reverse transcription kit (Qiagen) following the respective manufacturer's protocols. For real‐time quantitative PCR (RT‐qPCR), 22.5 ng of template cDNA, 2x QuantiNova SYBR Green PCR mix (Qiagen), and 300 nm of gene‐specific primer mix MSLN (forward (fw): CCT GAG GAC ATT CGC AAG TGG A; reverse (rev): CTT CCC TTC ACA AAG CGG TCG A, Merck) were used per 20 µL reaction, and each sample was prepared in triplicates. Reactions were run using the QuantStudio 3 Real‐Time PCR System (Thermo Fisher Scientific). Expression of the MSLN gene was normalized to the expression of the housekeeping gene β‐actin (fw: TGG AGC GAG CAT CCC CCA AA, rev: ATC ACC TCC CCT GTG TGG ACT) and the analysis was performed using the comparative delta CT method (ΔCT).

### 2.4 Mesothelin Peptides

Mesothelin peptides were obtained by microwave‐assisted solid phase synthesis following a standard Fmoc/tBu protection strategy using a Rink Amide ProTide LL resin (loading 0.18 mmol/g, 100–200 mesh) and N, N‐dimethylformamide (DMF) as the main solvent in a Liberty Blue automated synthesizer (CEM Corp., US) [24]. Fmoc removal cycles were performed with 20% v/v piperidine. Amino acid couplings were carried out with a 0.125  activating mix of N, N’‐diisopropylcarbodiimide (DIC) and ethyl 2‐cyano‐2‐(hydroxyimino)acetate (Oxyma). Palmitoylation of the peptide sequence was performed manually, after automated synthesis of the main sequence, by adding a mix of palmitic acid/DIC/oxyma (3/3/3 eq) in DMF for 1 h at RT, twice. Cleavage of peptide from the resin and global sidechain deprotection was achieved with a 95/2.5/2.5% v/v/v mix of trifluoroacetic acid/triisopropylsilane/miliQ water mix. Cleaved crude products were characterized by analytical RP‐HPLC and LC‐MS and purified by semi‐preparative RP‐HPLC. Detailed information regarding the amino acid sequences of the peptides is given in Table 1.

**TABLE 1:** Mesothelin Peptides used in this study.

Name	Sequence
MSLN1	Palm‐SLLFLLFSL‐CONH_2_
MSLN2	Palm‐ALPLDLLLFL‐CONH_2_
MSLN3	Palm‐PLTVAEVQKLLGPHV‐CONH_2_
MSLN4^*^	Palm‐PLTVAEVQKLLGPHVKKALPLDLLLFLKKSLLFLLFSL‐CONH_2_

* MSLN sequence integrated into the Mesovac formulation, termed MSLN4 comprises a combination of the three shorter MSLN peptide sequences (MSLN1, MSLN2, MSLN3), spaced by a cathepsin‐like cleavage motif (KK).

### 2.5 Mesothelin‐Nanovaccine (Mesovac) and Respective Control Formulations

Poly‐lactic‐co‐glycolic‐acid‐based (PLGA) NPs alone or with encapsulated adjuvants and MSLN4 peptide were prepared using an oil‐in‐water emulsion and solvent evaporation‐extraction method [25, 26]. Briefly, 50 mg PLGA was dissolved in 3 mL dichloromethane (DCM) with or without 5 µL pIC and 2 mg R848. The solution was added dropwise to 20 mL of aqueous 2% (w/v) polyvinyl alcohol (PVA) and emulsified for 60 s with 5 s rest each cycle using a sonicator (Sonifier 250, Branson, Danbury, USA). Following overnight evaporation of the solvents at 4°C, the NPs were collected by centrifugation (800 rpm for 30 min) at 4°C and redissolved in water. Subsequently, the NP solution was added dropwise to 20 mL of 1% homogenized chitosan oligosaccharide lactate solution and stirred at 4°C for 2 h. The coated NPs were finally collected by lyophilization.

### 2.6 Chemotherapeutic Drugs and PD‐L1 Antibody Formulation

To assess the efficacy of a combined treatment strategy with nanovaccine stimulation in PDAC PDOs, we formulated the following drug solutions based on the mentioned publications:

- Gemcitabine formulation (Gemcitabine^(R)^ 40 mg/mL): 840 nm gemcitabine [27];
- FOLFIRINOX formulation (FFX): a mixture of 5‐Fluorouracil (Medac^(R)^ 50 mg/mL; 37.6 µm), Irinotecan (Irinomeda^(R)^ 20 mg/mL; 16.9 µm), and Oxaliplatin (Medoxa^(R)^ 5 mg/mL; 7.9 µm) [28];
- Atezolizumab formulation (ATEZ): 0.1 mg/mL Atezolizumab (Atezolizumab Tecentriq^(R)^ 1200 mg/20 mL; 0.1 mg/mL) [29].

### 2.7 In Vitro Stimulation of PBMCs of Healthy Donors

Human peripheral blood mononuclear cells (PBMCs) were isolated from Leukocyte Reduction System (LRS) chambers received from 5 healthy HLA‐A^*^0201 donors at the local blood bank of the University Medical Center Göttingen (UMG ethical approval 29/07/23). After LRS processing, PBMCs were isolated by density gradient centrifugation utilizing SepmateTM tubes (Stemcell Technologies) containing Lymphocyte Separation Media (Density: 1077 g/mL) (Anprotec), following the manufacturer's instructions and as described before [20].

The resulting PBMCs were counted and seeded at a density of 1 × 10^5 cells per well in a 96‐well round‐bottom plate in X‐Vivo 15 medium (Lonza). A cytokine solution comprising 2000 IU/mL of GM‐CSF, 1000 IU/mL IL‐4, and Flt3‐Ligand (all Prepotech) was given to the medium of the cells to support dendritic cell stimulation.

Following overnight incubation, on the first day, 100 µL of the medium was aspirated and replaced with 100 µL X‐Vivo 15 medium containing an adjuvant solution combined with 1 µm non‐encapsulated mesothelin peptide (MSLN 2, MSLN 3, MSLN 2+3, or MSLN4), or with 15 µg/mL nanoparticles (PLGA, PLGA+Adj, or Mesovac). Additionally, unstimulated PBMCs as well as PBMCs receiving only the adjuvant solution and DMSO, were included as negative controls, while positive controls consisted of PBMCs stimulated with 2 µl/mL Concanavalin A (Con A, Invitrogen) and 27.5 µl/mL of Interferon‐γ (IFN‐γ) Capture Elisa peptide pool (ICE) (U‐CyTech). On day 2, 4 and 7, PBMCs were supplemented with a feeding solution of 20 IU/mL IL‐2, 20 ng/mL IL‐7, and 20 ng/mL IL‐15 (all from Prepotech), diluted in R10 media (containing 10 mm HEPES, 0.1 mg/mL Gentamicin, 1x Glutamax, and 10% human serum, diluted in RPMI 1640 medium), to facilitate T‐cell expansion. On day 9, PBMCs underwent re‐stimulation with 2 µg/mL of anti‐CD28 (BD Biosciences, Clone: CD28.2) and 2 µg/mL of anti‐CD49d (BD Biosciences, Clone: 9F10), diluted in R10 media and supplemented with mesothelin peptides, nanoparticles, or control reagents. After 1 h, 1x Brefeldin A (Biolegend) was added, and following a 4 h incubation, cells were immunophenotyped by flow cytometry. The gating strategy for IFN‐γ and TNF‐ɑ is described in the supplementary Figure S1. The protocol was performed based on the method published by Bozkus et al. [30].

### 2.8 PDAC Patient‐Derived T‐cells

PDAC patient‐derived T‐cells (referred to as PDAC T‐cells) were isolated from the tumor of an HLA^*^ 02:01 male patient with poorly differentiated PDAC and enriched in CTLs by flow cytometric cell sorting (FACS). Cells were isolated and expanded following the rapid expansion protocol as reported by Sorretino et al. [31]. Before co‐cultures with PDAC PDOs, PDAC T‐cells were cultivated in X‐VIVO medium supplemented with 600 IU/mL IL‐2 (Prepotech) overnight.

### 2.9 In Vitro Healthy and PDAC T‐cell Stimulation

#### 2.9.1 Preparation of Artificial Antigen‐Presenting Cells

The generation of artificial Antigen‐Presenting Cells (aAPCs) involved coupling HLA‐Ig dimers (BD Biosciences) and anti‐CD28 monoclonal antibodies (mAb, Biozol) to M‐450 Epoxy beads (Invitrogen). Following a protocol established by Chiu et al. [32], the initial step entailed washing 1 mL of M‐450 Epoxy beads with 0.1 m borate buffer. The beads were placed against a Dynal magnet MPC‐1 (Invitrogen) to remove the supernatant. Subsequently, the beads were resuspended in a mixture of 20 µg HLA‐A2 Ig dimer and 20 µg anti‐human CD28 mAb and incubated for 24 h on a rotator at 4°C. After two additional washing steps with 1 mL of bead wash buffer (containing 0.1 g of sodium azide, 2 mm EDTA, and 30 mL of human AB serum (Sigma), diluted in 956 mL of PBS without magnesium or calcium), the coupled beads were incubated again for 24 h at 4°C in fresh bead wash buffer. The successful formation of aAPCs was confirmed through flow cytometry analysis. For this purpose, 100 µL of aAPCs were stained with 1 µL of anti‐mouse IgG1‐PE, which recognizes the Fc portion of HLA‐A2‐Ig, and 1 µL of anti‐mouse IgG2a‐FITC (BD Biosciences), which identifies the Fc portion of anti‐CD28 mAb (Invitrogen).

For the loading of 1 µM of peptides onto aAPCs, following two PBS washes and adjustment to 10^7 beads/mL, different combinations were created: aAPCs coupled with pIC and R848 (aAPCs‐pIC‐R848), aAPCs coupled with MSLN4 (aAPCs‐MSLN4), and aAPCs coupled with pIC, R848, and MSLN4 (aAPCs‐pIC‐R848‐MSLN4). Uncoupled aAPCs (aAPCs) and aAPCs coupled with 1 mg/mL of Cytomegalovirus (CMV) pp65 (peptides & elephants) were used as negative and positive controls, respectively. After 3 days of MSLN peptide incubation at 4°C, aAPCs were prepared for co‐culture with either HLA^*^A2:01 T‐cells from an individual PDAC patient or from PBMCs from three healthy donors.

#### 2.9.2 Co‐Cultures of aAPCs with T‐cells from Healthy Donors and PDAC Patients

CD8+ T‐cells were isolated from PBMCs from healthy HLA^*^A2:01 donors, following the Miltenyi human CD8+ T‐cell isolation kit guidelines as per the manufacturer's protocol. Enrichment was confirmed through CD4/CD3/CD8 flow cytometric cell sorting (FACS) analysis. CD8+ T‐cells from PDAC patients were isolated from tumors as described in Section 2.8.

A total of 1 × 10^6 T‐cells were mixed with 1 × 10^6 peptide‐coupled aAPCs in a 1:1 ratio using T‐cell medium (5% human AB serum and 600 IU/mL of IL‐2 diluted in RPMI medium) and RPMI medium in a 6‐well plate. After 4 days of culture, cells received fresh TF 2x medium, and after 7 days, aAPCs were replaced by fresh peptide‐coupled aAPCs. After 3 weeks, the medium from stimulated T‐cells was collected for IFN‐γ quantification using ELISA. The T‐cells were used for immunophenotyping, ELISPOT, and co‐cultures with PDOs.

### 2.10 ELISPOT

IFN‐γ ELISPOT (BD) assay was performed according to the manufacturer's protocol. Briefly, 1 × 10^6 aAPCs were mixed with 1 × 10^6 stimulated T‐cells (2.10) in 200 µL of complete medium (RPMI 1640 with 10% FBS and 1% P/S) and stimulated for 24 h at 37°C, 5% CO_2_. Treatment with ICE peptide pool or aAPCs‐pp65 was used as a positive control, while treatments with only medium or with aAPCs were used as negative controls. Images from the wells were acquired using ImmunoSpot (Cellular Technologies).

Detection and counting of IFN‐γ+ cells in the ELISPOT assay images was done via a custom‐developed image analysis algorithm, which utilized adaptive thresholding and morphological operations to accurately locate the IFN‐γ+ cells. After isolating the dark spots (IFN‐γ+ cells), the algorithm applied connected‐component labeling and a size‐filtration step based on a predefined spot size range to eliminate artifacts from the true spots. Additionally, a comprehensive report was also generated, which lists out the count, size distribution, and additional metrics of the spots, which can be used for further statistical analysis. This code is available at https://github.com/ajinkya‐kulkarni/PyElispotAnalysis and as a web application at https://elispot‐analysis.streamlit.app/.

### 2.11 Organoid/Immune Cell Co‐Cultures

On day zero, fully matured organoids were harvested and subjected to mechanical dissociation using a 200 µL tip affixed to a 10 mL serological pipette. A single‐cell suspension was subsequently obtained by incubating the organoids for 3 min with Trypsin/EDTA (0.25%/0.02% (w/v) in PBS) solution at 37°C. Following this, a cell density of 1 × 10^4 cells was mixed with 15 µL of Matrigel and plated in 48‐well plates using a HOGM, as outlined in the protocol available at http://tuvesonlab.labsites.cshl.edu/wpcontent/uploads/sites/49/2018/06/20170523_OrganoidProtocols.pdf, with Y‐2732 supplementation.

On day 3, matured organoids were carefully collected using a 200 µL tip (tip end cut) attached to a 10 mL serological pipette and resuspended in an organoid passaging medium to remove the Matrigel. After 3 min centrifugation at 300xg, the medium was removed, and 20 µL of the passaging medium containing stimulated 1 × 10^5 human stimulated PDAC T‐cells was added.

To prepare the plate for co‐culture, 30 µL of 25% Matrigel (diluted in DMEM 1x) was added to each well of a 96‐well plate. After solidification of the Matrigel through a 15 min incubation at 37°C, 20 µL of the PDAC T‐cells‐PDOs mixture was added on top of the first Matrigel layer. Subsequently, an equivalent concentration of Matrigel was laid on top of the mixture. Following this, 200 µL of a 50% mixture of HOGM and 50% T‐cell culture medium was added on top of the solidified Matrigel layer. For the combinatorial treatment approach, different drug and antibody combinations as listed in Section 2.6 were added to the 50% OGM/ 50% T‐cell medium.

### 2.12 Knockdown of Mesothelin in PDAC PDOs

Fully matured organoids were dissociated into a single‐cell suspension as described in Section 2.11. The single‐cell suspension was then infected in a 25 µL Matrigel droplet (250 000 cells/droplet) at a multiplicity of infection (MOI) of 500. A vector containing scrambled shRNA (Scrambled; Vector ID: VB010000‐9496yha; VectorBuilder) was used as a control, and a Mesothelin knockdown vector (MesoKD; Vector ID: VB240722‐1232ymd; VectorBuilder) was used for experimental conditions; both vectors expressed mCherry. Once the droplet solidified, 500 µL of HOGM supplemented with 1:1000 Rho Kinase Inhibitor (Sigma) was added. Effective transduction of PDOs was confirmed by qPCR and flow cytometry (Figure S1) and by confocal imaging following organoid formation, based on mCherry expression. Transfected PDOs were then used for tumor‐on‐a‐chip experiments.

### 2.13 Validation of Mesothelin Nanovaccine on Microphysiological Systems

#### 2.13.1 Tumor‐on‐a‐Chip Design and Fabrication

Tumor‐on‐chip concept, design, and fabrication process were performed as described by Maulana et al. [33]. In brief, the tumor‐on‐chip consists of two polydimethylsiloxane modules (PDMS; Sylgard 184, Dow Corning, USA) featuring media channels and tissue chambers separated by an isoporous polyethylene terephthalate (PET) membrane (5 µm pore size: *r*_P_  =  5 µm; *ρ*_P_  =  6  ×  10^5^ pores per cm^2^; TRAKETCH PET 5.0 p S210  ×  300, SABEU GmbH & Co. KG, Northeim, Germany), coated with a glass‐like layer by a plasma‐enhanced, chemical vapor deposition (PECVD) process [34]. The tumor chambers (1 mm in diameter, 0.3 mm in height, six chambers per chip) branch off a main injection channel (0.2 mm in height) at a 45° angle upstream of a high‐resistance outlet channel and are located right below the medium channel featuring a height of 200 µm. The 5 µm pore size allows T‐cell trafficking and passive diffusion of diluted species [35]. The two PDMS chip modules are produced by mixing the elastomer base and curing agent in a 10:1 mass ratio, followed by degassing and pouring onto silicon wafer master molds for replica molding of the microstructures. After curing of the PDMS (60°C for respectively 2 or 4 h), the microfluidic layers were assembled in three consecutive bonding steps: (i) tumor layer to glass coverslip, (ii) medium layer to the membrane, (ii), and (iii) the tumor layer to the medium layer. In all steps, bonding was achieved by oxygen plasma activation (75 W, 20 sccm O_2_; Diener Pico, Diener electronic GmbH + Co. KG, Ebhausen, Germany) for 18 s. Bonded parts were baked at 60°C for 1 h after each bonding step, and overnight after the entire chip was assembled. All chips were O_2_‐plasma treated (75 W, 20 sccm O_2,_) for 5 min to sterilize and hydrophilize the PDMS surface before cell injection. A representative image of the tumor‐on‐chip is shown in Figure S2.

#### 2.13.2 PDAC PDOs Seeding on Chip

After chip sterilization and hydrophilization with O_2_‐plasma, the channels were flushed using a 100 µL pipette with 70% ethanol followed by rinsing three times with PBS. The chips were kept at RT until cell seeding. The PDAC PDOs were seeded into the chip one day before T‐cell perfusion.

PDAC PDOs were resuspended in dextran hydrogel supplemented with 0.5 mmol/L RGD peptide. Once the PDAC PDOs were mixed with the hydrogel, 10 µL was immediately injected into each chip via the tumor inlet, followed by plugging the inlet and outlets of the injection channel with a 0.7 mm diameter metal plug (45473; Menzanium), and the injection channel was flushed via negative pressure to remove any remaining cells. Then, plugs from the medium channel inlet and outlet were carefully removed and inserted into the tumor channel inlet and outlet. The medium channel was subsequently flushed three times with 100 µL of cell‐specific culture medium to remove any deposited hydrogel. The chips were then incubated at 37°C, 5% CO_2,_ and 95% rH for 30 min to allow hydrogel crosslinking.

#### 2.13.3 T‐Cell Perfusion and Chip Culture

To prepare cells for fluorescence labelling, the T‐cell pellet was resuspended in 5 µM of Biotracker Cystine‐FITC Live Cell Dye (SCT047; Sigma–Aldrich) dissolved in T‐cell culture medium and incubated for 15 min at 37°C, 5% CO_2,_ and 95% rH. The cells were then washed twice by adding PBS 1x up to 10 mL and centrifuging at 300 x g for 8 min at 8°C for each washing cycle. Afterward, the final cell pellet was resuspended in T‐cell culture medium.

The day of T‐cell perfusion was defined as day 0 for all experiments. Starting from day ‐1, the chips were connected to constant medium perfusion via an external syringe pumping system (LA‐190, Landgraf Laborsysteme HLL GmbH, Langenhagen, Germany). The chips were connected to the syringe pump using blunt 21 GA stainless steel needles (made from the dispensing needles by removing the plastic hub after dissolving the glue overnight in a 70% ethanol solution) connected to Tygon tubings (0.51 mm inner diameter, Tygon ND 100–80 Medical Tubing, Saint‐Gobain Performance Plastics Pampus GmbH, Willich, Germany), 21 GA stainless steel plastic hub dispensing needles (e.g., KDS2112P, Weller Tools GmbH, Besigheim, Germany) and Luer Lock style syringes. For T‐cell perfusion through the medium channel, the inlet of the channel was equipped with a 100 µL‐pipette tip reservoir holding 400 µL of T‐cell suspension (200 000 T‐cells/chip). Once T‐cells were suspended in the pipette tip reservoir, the flow rate was set to 20 µL/h in pump withdrawal mode and transferred into an incubator at 37°C, 5% CO_2,_ and 95% rH. After 20 h, the perfusion setup was changed to push mode to have co‐culture medium dispensed from the syringes to a collecting reservoir. The chips were perfused linearly in this mode at a flow rate of 20 µL/h until day 7. Medium in the syringes was refilled after 2 days of perfusion. Atezolizumab and FOLFIRINOX treatment was performed by supplementing 50% OGM/ 50% T‐cell medium according to the concentration described in Section 2.6, from day 1 to day 7.

#### 2.13.4 Imaging Acquisition and Analysis

The quantification of T‐cell infiltration in PDAC organoids was performed using z‐stack images acquired with a spinning disk confocal microscope (Cell Observer Z1, Carl Zeiss) and an EC Plan‐Neofluar 10X/0.30 objective (Zeiss) (Figure S3). Image analysis was carried out with Imaris software, version 9.5 (Oxford Instruments). T‐cell segmentation was performed using the Spot detection tool, with the XY diameter set to 5 µm and the Z height to 10 µm. PDAC organoid volume was determined using the Surfaces function tool. This 3D segmentation enabled the identification of each T‐cell and PDAC organoid as individual objects inside every tissue chamber. Once segmentation was established, simple measurements such as shortest distances to surfaces (Filter tool) were obtained according to specific criteria. For T‐cell infiltration, only T‐cell objects with surfaces at a minimum distance of 0 µm (up to a maximum of –200 µm, i.e., T‐cells located within the PDAC organoid at a depth of up to 200 µm from the surface) from PDAC organoid surfaces were considered, allowing the quantification of T‐cells that were either in contact with or infiltrated into PDAC organoids. For all measurements, image data from a minimum of 2 chips were pooled for each condition for further statistical analysis.

### 2.14 ELISA

IFN‐γ levels were measured by ELISA, using an ELISA MAX Deluxe Set (Biolegend). In brief, Immuno plates (Nunc) were coated with a 1:1000 dilution of anti‐human IFN‐γ antibody and incubated overnight at 4°C. Following this step, plates were washed twice with a washing buffer (PBS with 0.05% Tween 20), and then blocked with 1x Assay Diluent A for 1 h at RT. Next, the plates were rewashed and incubated with 100 µL of the supernatant from the respective experiments for 2 h at RT. After rewashing, a detection antibody was added, followed by another round of washing. Next, the HRP (horseradish peroxidase) enzyme was added, and the plates were rewashed. Subsequently, the plates were incubated with an enzyme‐substrate solution for 20 min at RT and stopped by adding 100 µL of a 2 N sulfuric acid solution. The optical density (OD) at 450 nm was measured using an automated plate reader BioTek (EON).

### 2.15 Flow Cytometry Analysis

For immunophenotyping of the human PBMC stimulation and confirmation of MSLN knockdown, up to 10^6 cells were resuspended in 100 µL FACS buffer (PBS, 1% BSA) per staining, transferred to flow cytometry tubes, and centrifuged at 300xg for 5 min. The pellets were stained with 1 µL of Zombie UV (Biolegend) diluted in 100 µL of PBS. After 10 min incubation in the dark, cells were washed twice with 2 mL of FACS buffer and centrifuged at 300 x g for 5 min. The pellets were then incubated for 10 min in 95 µL FACS buffer and 5 µL human FcR Blocking Reagent (Biolegend). For extracellular staining of CD3/CD4/CD8 or MSLN, 0.5 µL of each antibody (see Table S1 and Mesothelin K1‐PE, sc‐33672, Santa Cruz Biotech) was added. Cells were then washed twice with FACS buffer and incubated with fixation buffer (Invitrogen) in a dilution of 1:1 with FACS buffer for 10 min. Intracellular staining of IFN‐γ and TNF‐ɑ was performed following two wash steps with 1x permeabilization solution (Biolegend) diluted in water (Perm/ Wash buffer), with 0.5 µL antibody each (see Table S1), for 30 min. As a final step, cells were washed twice with the Perm/ Wash buffer and resuspended in a 400 µL FACS buffer. Samples were measured on a BD FACS CANTO II, and data analysis was performed with FlowJo software. The gating strategy for human PBMC reactivity and MSLN expression in transfected PDAC PDOs is shown in Figures S4 and S5, respectively.

For flow cytometric immunophenotyping of the PDAC T‐cell stimulation, PDOs and PDAC T‐cells from PDAC T‐cell/PDO co‐cultures were resuspended in 1x PBS/0.5% BSA/ 2 mM EDTA (PEB) buffer. Up to 10^6 cells were resuspended in 100 µL per staining, transferred to a 96‐well plate, and centrifuged at 300 x g for 5 min. The pellets were resuspended in 35 µL PEB buffer, and 5 µL human FcR Blocking Reagent (Miltenyi Biotec) was added to each well. Samples were mixed, followed by the addition of the staining antibodies (1 µL each; Table S2), then remixed and incubated for 10 min at 4°C. Unbound antibodies were removed by washing twice with 250 µL PEB buffer. The pellets were then resuspended in a 150 µL PEB buffer. For identification of dead cells, propidium iodide (1 µg/mL) was added immediately before sample acquisition. Samples were acquired on a MACSQuant Analyzer 16, and data analysis was performed with MACSQuantify software (both Miltenyi Biotec). Gating strategies for PDAC T‐cell selection, characterization of PDAC T‐cells, and PDAC PDOs from the co‐cultures are shown in Figures S6–S8, respectively.

### 2.16 Statistical Analysis

Statistical analysis was performed with GraphPad Prism 9. All presented data are expressed as means with corresponding standard errors of the means (SEMs). Comprehensive information regarding the significance tests, including the number of replicates and associated *p*‐values, can be found in the respective figure legends.

## 3 Results

### 3.1 Workflow for In Vitro Nanovaccine Evaluation in PDAC PDO/T‐Cell Co‐Cultures

**FIGURE 1:**
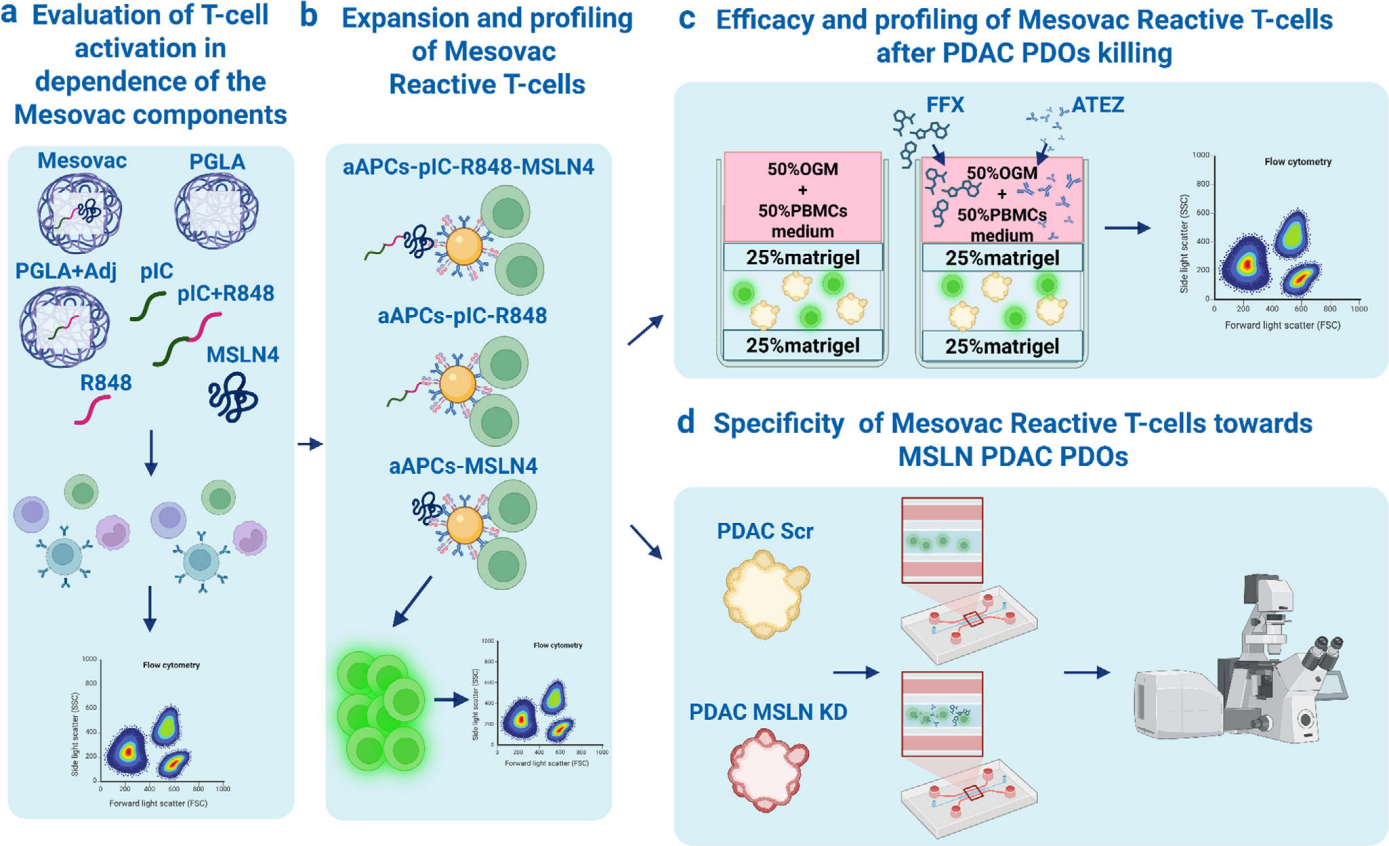
Workflow for in vitro evaluation of the MSLN nanovaccine formulation. (a) Individual components and their combinations within the Mesovac formulation were assessed for their capacity to elicit immune responses in healthy PBMCs. (b) Reactive Mesovac‐stimulated PDAC T‐cells were expanded and phenotyped via flow cytometry. (c) Expanded PDAC T‐cells were co‐cultured with PDAC PDOs to assess their cytotoxic activity. (d) The antigen specificity of Mesovac‐induced T‐cells was evaluated using a tumor‐on‐a‐chip model by quantifying T‐cell infiltration into PDAC PDOs with high (PDAC Scr) or low (PDAC MSLN KD) MSLN expression. Scr = scrambled; KD = Knockdown. Created with BioRender.com.

To evaluate the efficacy of the MSLN‐based nanovaccine (Mesovac), we developed a comprehensive in vitro pipeline (Figure 1), consisting of the following key steps:

- T‐Cell Activation Assessment: We evaluated the ability of individual Mesovac peptides, as well as their combinations, to induce cytokine production from peripheral blood mononuclear cells (PBMCs) using flow cytometry. This step assesses the immunostimulatory potential of each component.
- Expansion and Profiling of PDAC‐derived T‐Cells: To characterize and expand the population of PDAC‐derived T‐cells that respond to the complete Mesovac peptide pool, we utilized artificial antigen‐presenting cells (aAPCs). This approach reduces variability associated with natural APCs and enables more controlled T‐cell expansion and analysis.
- Cytotoxicity against PDAC PDOs: We tested the ability of Mesovac‐reactive T‐cells to kill HLA‐A^*^02:01‐matched PDAC PDOs, both alone and in combination with standard therapies, including FOLFIRINOX (FFX) and Atezolizumab (ATEZ).
- Specificity Analysis in the Tumor‐on‐a‐Chip Model: To evaluate the antigen‐specific killing of PDAC PDOs, Mesovac‐reactive human T‐cells were co‐cultured with PDOs expressing high or low levels of MSLN within a complex tumor‐on‐a‐chip system. This setup enables high‐resolution assessment of therapeutic specificity in a physiologically relevant microenvironment.

### 3.2 Mesothelin‐Based Nanovaccine Formulation to Target PDAC

To validate the feasibility of this human organoid–immune cell co‐culture approach to evaluate cancer nanovaccines in vitro, we used a Mesothelin (MSLN) based nanovaccine as a use case. MSLN, a known cell surface glycoprotein highly expressed in PDAC, has been extensively explored as a target for immunotherapeutic strategies [7]. Acknowledging the significance of a dual adjuvant strategy in stimulating robust anti‐tumor responses [36, 37], we selected Toll‐like receptor (TLR) TLR3 ligand poly (I:C) (pIC) and TLR7/8 ligand resiquimod (R848) for the nanovaccine formulation due to their synergistic promotion of dendritic cell (DC) immunostimulation [38]. PLGA was chosen as the encapsulating agent to contain the MLSN antigen alongside with the two adjuvants (Figure S9). This choice was motivated by PLGA's capacity to provide a controlled release system with low toxicity and high biocompatibility [39]. Considering the ability of extended peptide sequences to enhance both CD4+ and CD8+ responses and to encompass a wider spectrum of HLA types [40], the MSLN sequence, integrated into the Mesovac nanovaccine formulation, termed MSLN4 (Table 1), comprises a combination of three shorter MSLN peptide sequences (MSLN1, MSLN2, MSLN3). These peptides are known to be immunogenic and stimulate T‐cells that are associated with increased survival in PDAC patients [41]. In a PDAC mouse model, we could already show that this formulation was able to significantly reduce KPC tumor progression, including metastasis [42]. Using the workflow described above, the efficacy of Mesovac was compared with both controls, PLGA alone (PLGA) and the combination of the two adjuvants encapsulated in PLGA (PLGA‐Adj) (Figure S9).

### 3.3 MSLN Expression Profiles in PDAC PDOs

**FIGURE 2:**
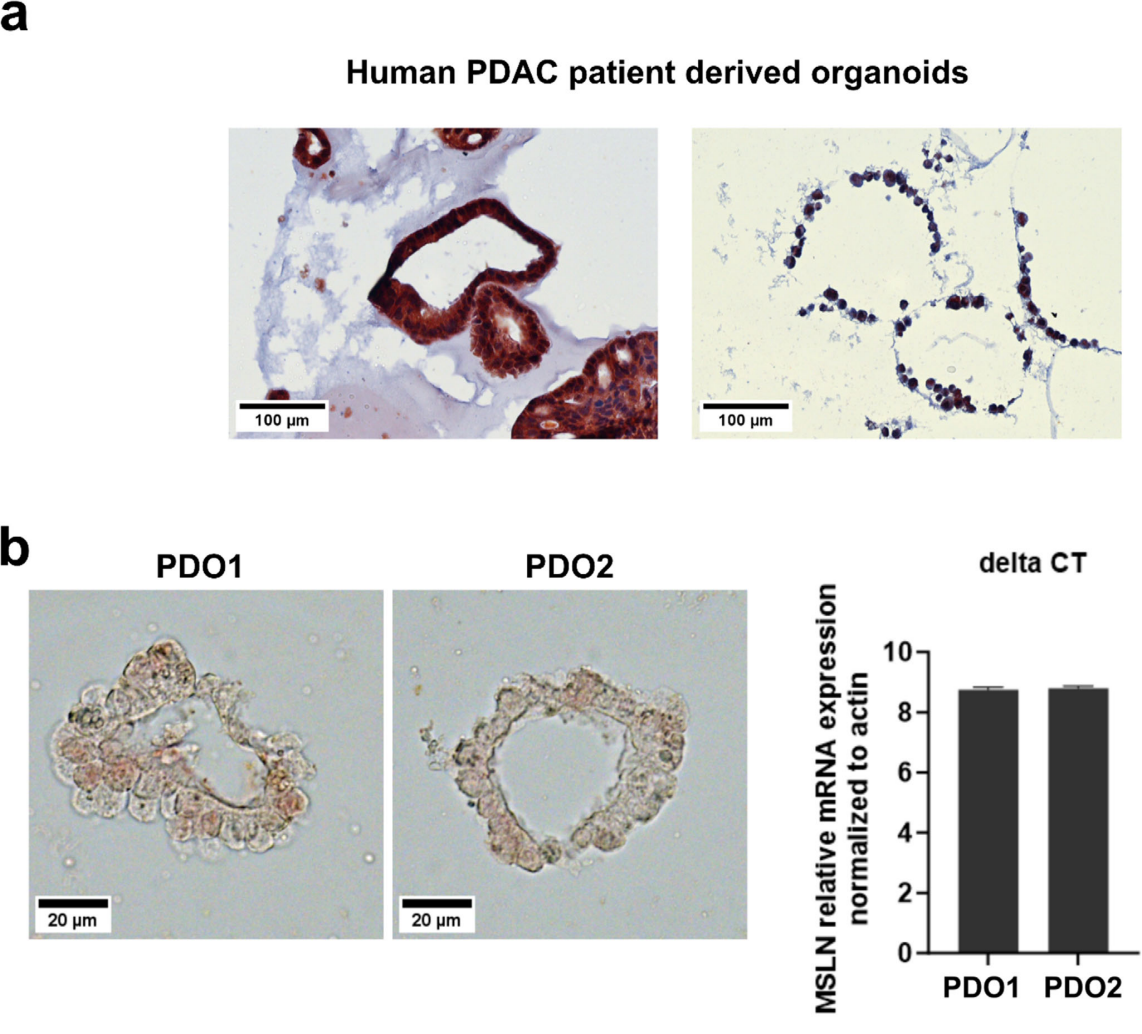
Confirmation of MSLN expression in PDAC patient‐derived PDAC organoids (PDOs). (a) Representative IHC staining of MSLN protein expression in PDOs derived from two different PDAC patients demonstrating distinct expression levels of MSLN in each PDO. (b) Representative IHC staining of MSLN protein expression (left panel) and qPCR analysis (right panel) confirmed similar levels of MSLN gene expression in PDO1 and PDO2, used for our therapy efficacy studies. Scale bars represent 100 µm in (a) and in 20 µm (b).

Drawing from documented literature highlighting the increased expression of mesothelin in PDAC patients [43, 44], we first verified the protein expression of mesothelin in HLA‐A02:01 PDAC PDOs selected for this study. As anticipated, PDOs exhibited varying patterns of MSLN expression (Figure 2a and data not shown), reflecting inter‐patient variability. For our therapy efficacy study, we selected two HLA A2^*^01 matched organoids (PDO1 and PDO2) with comparable MSLN gene expression, as confirmed by qPCR analysis (Figure 2b). The mutational profiles were assessed for both organoids by using an RNA sequencing (RNA‐seq) protocol, revealing mutations in *KRAS, TP53, and SMAD4* in both PDOs (Table S3), consistent with the mutations described in PDAC primary tumors [45].

### 3.4 Mesovac Shows a Trend to Increase IFN‐γ+ T‐Cells from Healthy PBMC Donors

**FIGURE 3:**
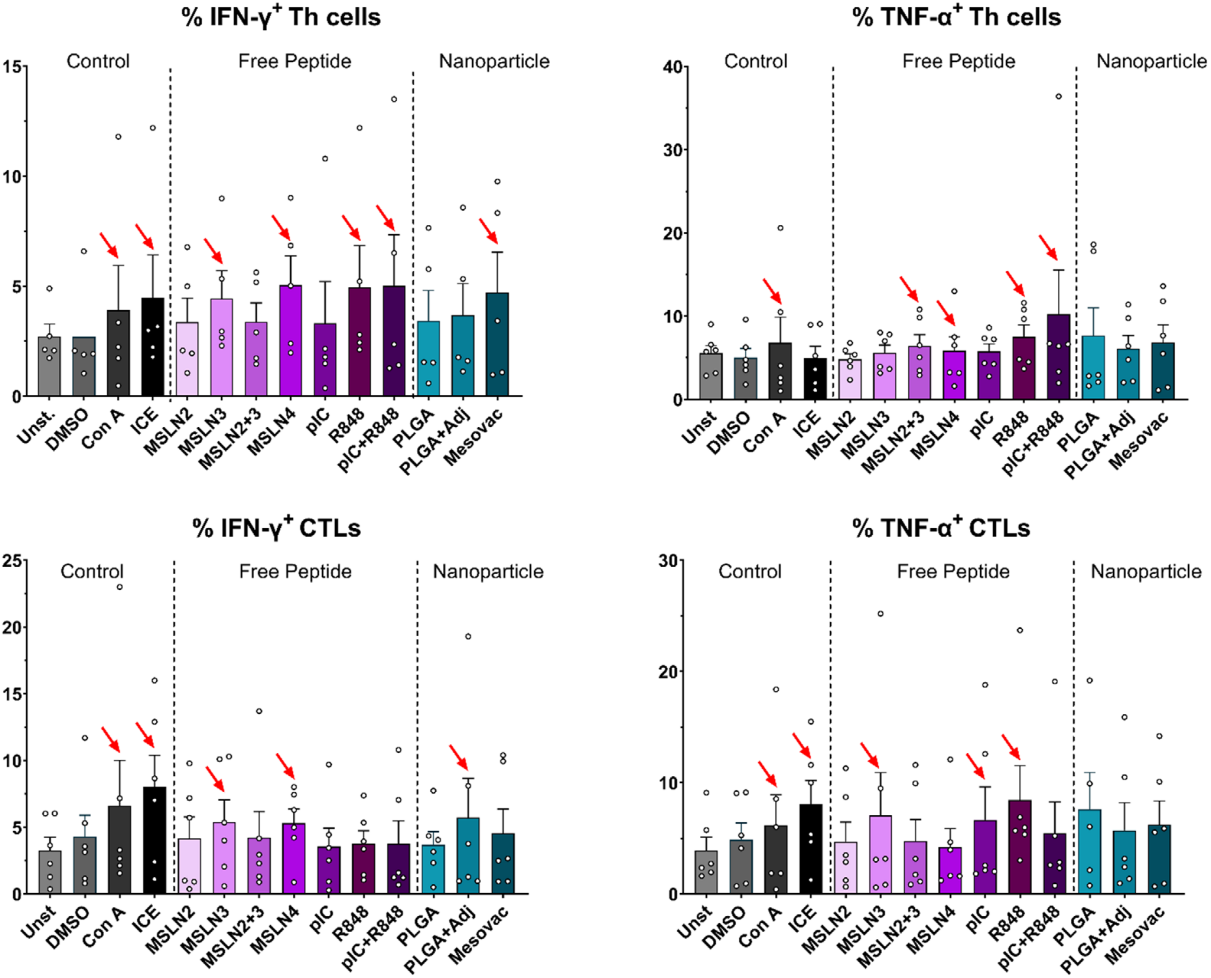
MSLN3 and MSLN4 peptides or Mesovac lead to a minor stimulation of IFN‐γ producing T‐cells. Reactivity of Th cells and CTLs was quantified by IFN‐γ and TNF‐α levels. Unstimulated cells (Unst.) and DMSO‐stimulated cells were used as negative controls, while Con A and ICE stimulation served as positive controls. Data represent the mean ± SEM of five independent human PBMCs. Red arrows indicate an increase of respective T‐cells in comparison to unstimulated T‐cells (Unst).

As a first step of our proposed workflow, we assessed an effective immune response following the treatment with the nanovaccine Mesovac by conducting an in vitro T‐cell‐based peptide stimulation protocol using unfractionated PBMCs from 5 healthy donors as described by Bozkus et al. [30] (Figure S10). A 9‐day stimulation period was performed with various stimuli, including the free peptides MSLN2, MSLN3, a combination of MSLN2 and MSLN3 (MSLN 2+3), and MSLN4, the adjuvants pIC or R848 alone, or a combination of pIC and R848, as well as Mesovac and its respective controls (PLGA, PLGA+Adj). The PBMCs were analyzed using flow cytometry to quantify the responses of T helper (Th) cells and cytotoxic T lymphocytes (CTLs) based on cell count and the intracellular expression of IFN‐γ and TNF‐α (Figure 3). Stimulation with Concanavalin A (Con A) and the ICE peptide pool was used as positive controls and showed the expected unspecific increase of T‐cells of about 10%–20%. Following stimulation with either free peptides or the nanoparticles containing Mesovac, we did not observe any substantial changes in the overall number of T‐cells (% viable CD3+ T‐cells), CTLs (% viable CD3+/CD8+ T‐cells), and Th (% viable CD3+/CD4+ T‐cells) cells (Figure S11). Although not statistically significant, a trend toward a higher percentage of IFN‐γ+ CTLs and IFN‐γ+ Th cells was noticeable when PBMCs were stimulated with either the MSLN3 or the MSLN4 peptides alone. Furthermore, the nanovaccine Mesovac led to a higher percentage of IFN‐γ+ Th cells than the stimulation with its respective controls. As for TNF‐α+ cells, we only detected a minor increase in response to MSLN3 peptide. Consistent with previous studies [38], a general increase in IFN‐γ+ and TNF‐α+ cells was observed when PBMCs were stimulated with adjuvants alone. However, in the case of combinations of adjuvant peptides (pIC+R848) or PLGA+Adj, an additional increase in the percentage of Th+ TNF‐α+ and CTLs+ IFN‐γ+ T‐cells was observed. In summary, despite substantial variability among donors, our observations indicate that the individual components of the Mesovac formulation, when used independently, induced a notable proportion of reactive T‐cells. Moreover, the Mesovac combination demonstrated an ability to increase IFN‐γ+ T‐cells in vitro. Although this increase was not significant with our limited number of PBMC samples tested, the modest increase we see in IFN‐γ+ T‐cells is in line with results seen after prophylactic vaccine or peptide stimulation with unexpanded PBMCs from non‐immunized donors [46]. This in vitro approach shows the potential of this step to infer the reactivity of each component of the nanoparticles, alone or in combination.

### 3.5 MSLN4‐pIC‐R848‐coupled aAPCs Induce and Expand Reactive T‐Cells

**FIGURE 4:**
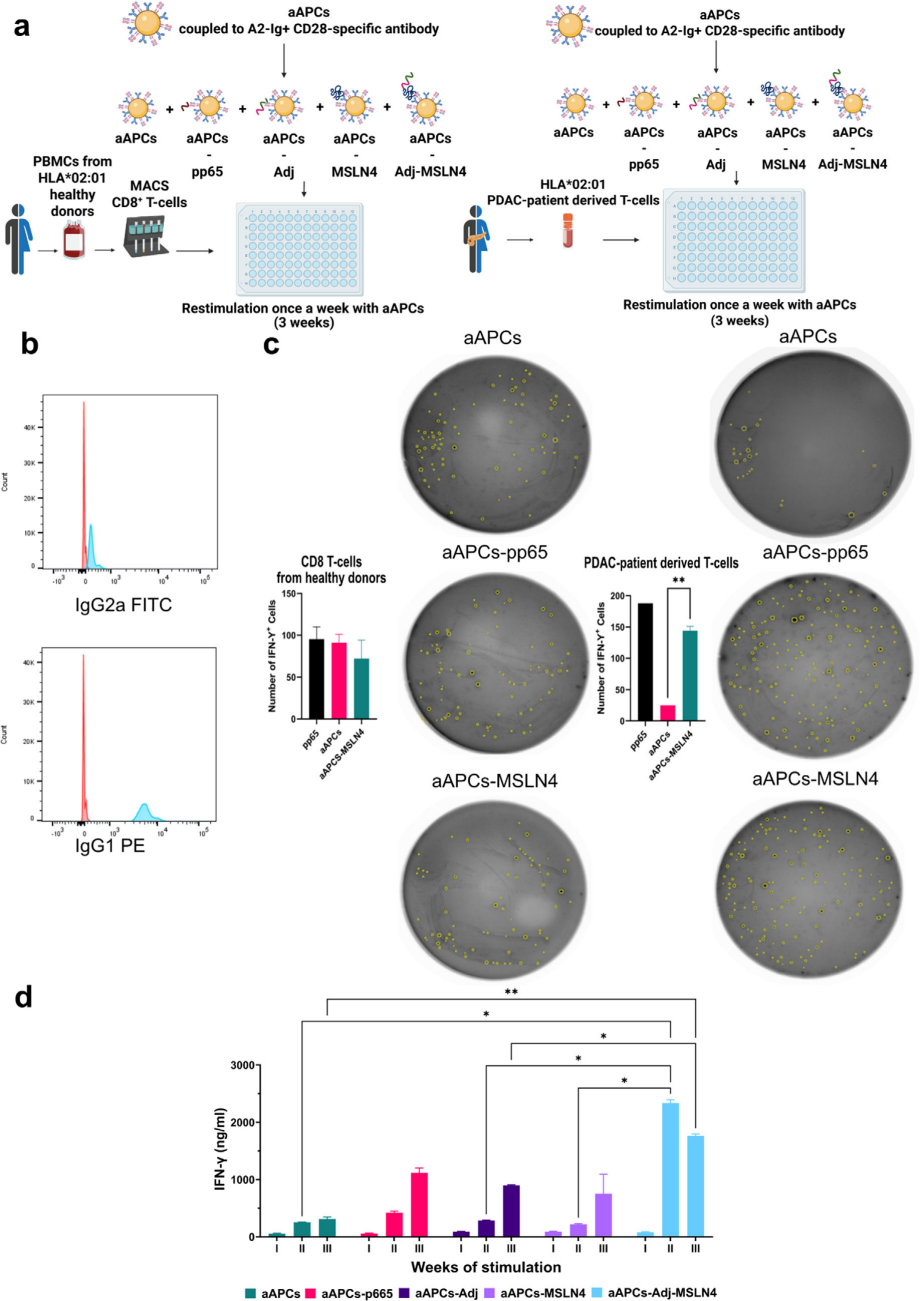
Enhanced activation of PDAC patient‐derived T‐cells upon stimulation with pIC‐R848‐MSLN4‐coupled aAPCs. (a) Ex vivo activation protocol for T‐cells obtained from healthy donors (left scheme) and from a tumor of a PDAC patient (right scheme). T‐cells were co‐cultured with aAPCs coupled with either both pIC and R848 (aAPCs‐pIC‐R848), or MSLN4 alone (aAPCs‐MSLN4), or with the combination of adjuvants together with MSLN4 (aAPCs‐pIC‐R848‐MSLN4). aAPCs coupled with pp65 peptide (aAPCs‐pp65) or uncoupled aAPCs (aAPCs) served as positive and negative controls, respectively. A 3‐week stimulation with the respective aAPCs in round‐bottom 96‐well plates resulted in the expansion of antigen‐induced human T‐cells. Created with BioRender.com. (b) Generation of aAPCs, formed by coupling HLA‐A2‐Ig and anti‐CD28 to beads, was confirmed by flow cytometry showing positive staining with anti‐IgG2a‐FITC and anti‐mouse IgG2a‐FITC, which identifies HLA‐A2‐Ig and CD28, respectively. (c) ELISPOT quantification of IFN‐γ‐positive T‐cells from healthy donors (left panel) and patient‐derived T‐cells (right panel) after 3 weeks of stimulation with different non‐ and peptide‐loaded aAPCs, demonstrating increased activation of PDAC patient‐derived T‐cells with aAPCs‐MSLN4. Representative images of ELISPOT membranes show IFN‐γ‐positive T‐cells in yellow from co‐cultures with aAPCs (without peptide coupling) as control, or aAPCs‐pp65, or aAPCs‐MSLN4. (d) ELISA quantification of IFN‐γ levels in the medium from co‐cultures of indicated peptide‐coupled aAPCs with PDAC patient‐derived T‐cells after one (I), two (II), and three weeks (III) is shown. Significant enhanced IFN‐γ levels were observed in co‐cultures with aAPCs‐pIC‐R848‐MSLN4 after two weeks of stimulation, compared to aAPCs stimulation alone. ELISPOT and ELISA data represent the mean ± SEM of two technical replicates: ^*^ *p* < 0.05, ^**^ *p* < 0.01 (One‐way‐ANOVA followed by Dunnett's; Two‐way ANOVA followed by Dunnett's comparisons test for ELISA analysis).

The stimulation and expansion of T‐cells with natural APCs such as dendritic cells (DCs) is challenging and leads to a large variability in the quality and quantity of the DCs obtained from leukapheresis. Moreover, activation of T‐cells with CD3 antibodies is associated with a decrease in antigen specificity and does not sustain long‐term growth of CTLs [47]. To address these limitations in expanding antigen‐stimulated T‐cells, we implemented an ex vivo induction and expansion method for HLA^*^02:01 T‐cells. These T‐cells were derived from i) PBMCs from healthy donors and ii) from a tumor of a PDAC patient (Figure 4a). T‐cells reactive to Mesovac components were achieved through co‐cultures with HLA‐Ig‐coated aAPCs, as established by Oelke et al. [47]. Following this method, we conducted three rounds of enriching healthy CD8+ T‐cells (Figure 4a, left scheme) and PDAC patient‐derived T‐cells (Figure 4a, right scheme) with aAPCs coupled with either adjuvant combination (aAPCs‐pIC‐R848), MSLN4 (aAPCs‐MSLN4), or the complete set of Mesovac constituents (aAPCs‐pIC‐R848‐MSLN4). Uncoupled aAPCs (aAPCs) and aAPCs‐coupled with the CMV peptide pp65 (aAPCs‐pp65) served as negative and positive controls, respectively.

The aAPCs consist of a dimeric form of HLA (HLA‐Ig) coupled with CD28‐specific antibodies, representing co‐stimulatory molecules. Successful coupling of peptides and adjuvants to the aAPCs was confirmed by flow cytometry. We could show a discernible shift in the corresponding antibody staining (blue peak), clearly distinguishing it from beads (red peak) alone (Figure 4b). Following the coupling of peptides to aAPCs, co‐cultures were performed either with T‐cells from healthy donors or with T‐cells from a PDAC patient. At the end of each week of stimulation (I, II, III), the T‐cell response to the peptide‐coupled aAPCs was determined in 2 ways: (i) medium from the co‐cultures was collected, and IFN‐γ production from T‐cells was measured by ELISA, and (ii) the number of IFN‐γ secreting T‐cells was determined by ELISPOT. After three rounds of coupled‐aAPCs stimulation, the anti‐IFN‐γ+ incubated ELISPOT membranes captured a significant higher number of IFN‐γ+ cells from the PDAC patient‐derived T‐cells co‐cultured with MSLN4‐coupled aAPCs compared to uncoupled aAPCs (Figure 4c, right). In contrast, T‐cells from healthy donors did not show a significant response after MSLN4‐coupled aAPCs co‐culture (Figure 4c, left). As depicted in Figure 4d, PDAC reactive T‐cells showed a general increase in the production of IFN‐γ after each week of re‐stimulation with coupled‐aAPCs. Notably, higher T‐cell reactivity was observed when co‐cultured with aAPCs‐pIC‐R848‐MSLN4, especially evident after two weeks of re‐stimulation.

**FIGURE 5:**
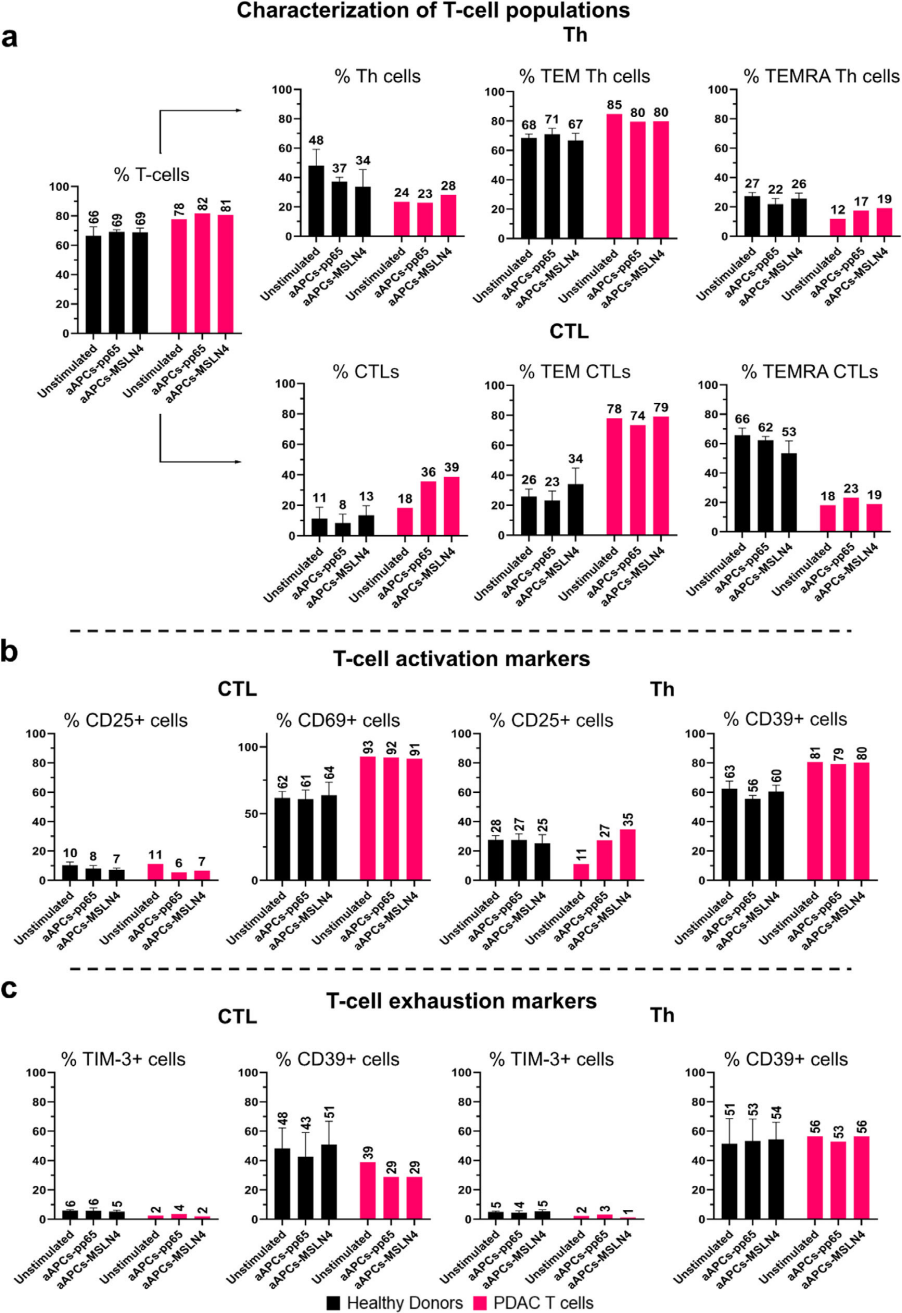
aAPCs‐MSLN4‐stimulation elicits a functional population of Th TEMRA cells in PDAC patient‐derived T‐cells. (a) The assay, conducted by flow cytometry, involved three weeks of stimulation with aAPCs‐MSLN4, aAPCs alone (negative control), or aAPCs‐pp65 (positive control), to assess healthy and PDAC patient‐derived T‐cells, subsequently classified into CTLs and Th cells. These cell populations were further phenotyped to distinguish between TEM and TEMRA T‐cells by flow cytometry. (b) To evaluate the functional capacities of Th and CTL cells, antibodies against activation markers such as CD25 and CD69 were employed in flow cytometry to stain healthy and PDAC patient‐derived T‐cells. (c) T‐cells exhibiting positivity for TIM‐3 and/or CD39 were considered as exhausted T‐cells. The data presented in the figure represent the mean ± SEM of samples obtained from three healthy T‐cell donors and one T‐cell donor, the PDAC patient.

To further delineate the specific subpopulation of T‐cells responsible for the increased reactivity upon binding to aAPCs coupled with MSLN4, we categorized T‐cells into distinct subsets, Th cells and CTLs. Given the dynamic nature of T‐cell differentiation, we further stratified Th cells and CTLs into “effector” memory (TEM) and “terminally differentiated” effector memory (TEMRA) cells. These subsets exhibit a more cytotoxic profile, yet TEMRA cells display increased expression of exhausted genes compared to TEM cells [48]. Important to note is that our study utilized T‐cells derived from a tumor from a single patient with PDAC. Upon stimulation with aAPCs‐MSLN4, we observed an increase in both Th cells (4%) and CTLs (21%), while there was only a marginal 3% increase in the overall T‐cell count compared to uncoupled aAPCs stimulation (Figure 5a). Specifically within Th cells, we noted a shift toward a TEMRA‐associated profile, evidenced by a 7% increase in TEMRA Th cells accompanied by a 5% decrease in TEM Th cells (Figure 5a). In contrast, although there was a substantial increase in the general CTL population (twice as much as in unstimulated cells) with aAPCs‐MSLN4, this increase did not arise from an increase in TEM CTLs or TEMRA CTLs. We found no differences in the amount of CTLs and Th cells expressing the activation marker CD69 (Figure 5b) or the exhaustion marker TIM‐3 (Figure 5c). However, upon stimulation with aAPCs‐MSLN4, a higher percentage of Th cells expressing the activation marker CD25 and a reduction of the percentage of Th cells positive for CD39, a marker for exhaustion (albeit not significant) was found. These results indicate that aAPCs‐MSLN4‐stimulated T‐cells are activated and not exhausted. By contrast, CTLs showed no significant differences in TEM or TEMRA subsets between aAPCs and aAPCs‐MSLN4‐stimulated T‐cells from the PDAC patient. Taken together, these findings suggest that the increased reactivity of PDAC patient‐derived T‐cells upon stimulation with MLSN4‐coupled aAPCs might predominantly originate from Th cells exhibiting a TEMRA phenotype with low expression of exhaustion markers. Interestingly, healthy T‐cells presented an 8% increase in TEM‐positive CTLs, along with a 13% decrease in TEMRA‐positive cells when aAPCs and aAPCs‐MSLN4 stimulated T‐cells were compared. In summary, the application of this system did not only enable the ex vivo induction and expansion of MSLN4‐stimulated PDAC patient‐derived T‐cells, demonstrating stronger reactivity when exposed to the constituents of the nanovaccine Mesovac, but also facilitated a thorough characterization of the expanded T‐cell profile.

### 3.6 aAPC‐Adj‐MSLN4‐stimulated PDAC Patient‐Derived T‐Cells Specifically Infiltrate PDAC Patient‐Derived Organoids Expressing MSLN

**FIGURE 6:**
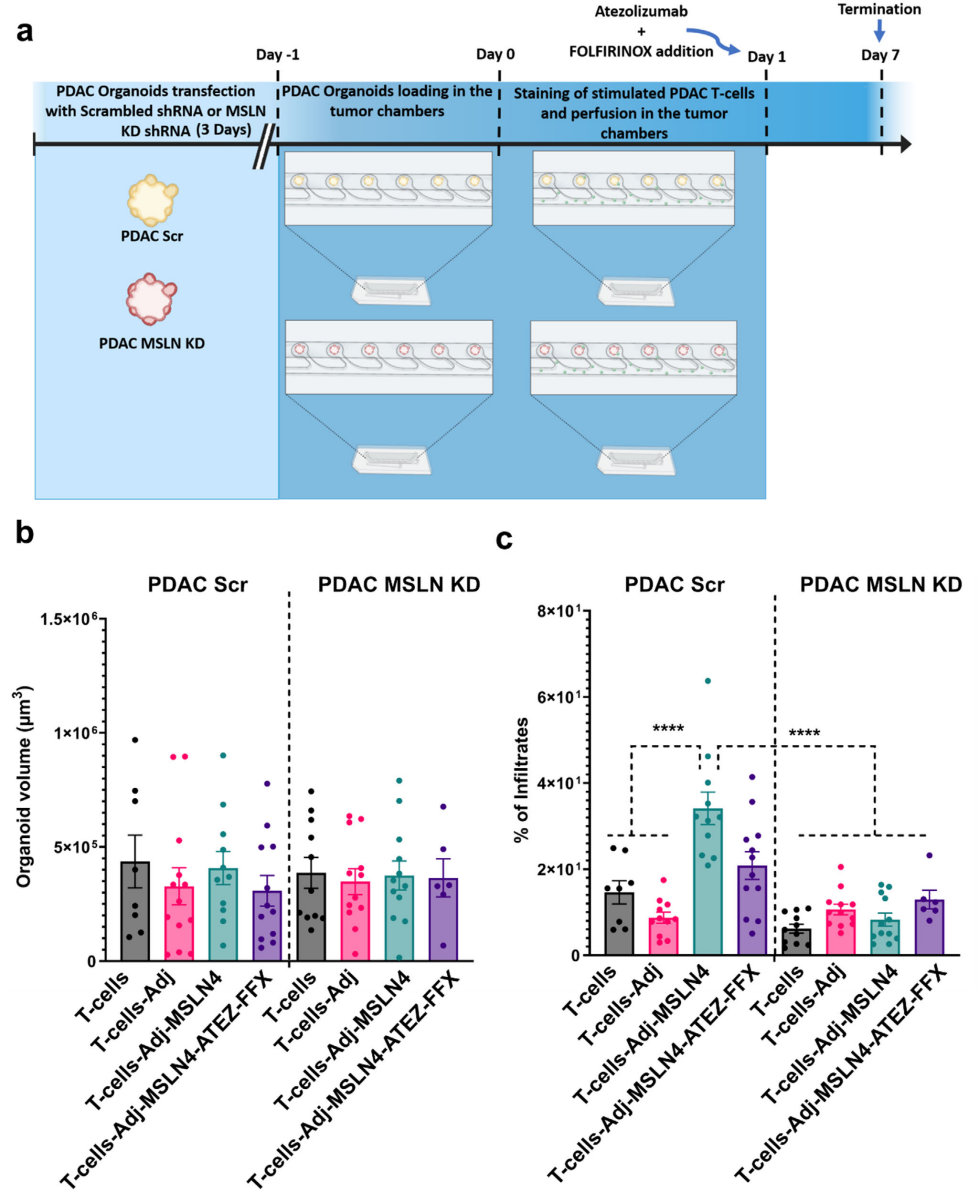
Enhanced infiltration of specific Adj‐MSLN4–stimulated PDAC patient‐derived T‐cells into PDAC PDOs, with or without Atezolizumab plus FOLFIRINOX. (a) Schematic overview of the tumor‐on‐a‐chip co‐culture experiments. Created with BioRender.com. (b) Volume of wild‐type PDAC PDOs (PDAC Scr) or MSLN knockdown PDAC PDOs (PDAC MSLN KD) after 7 days of co‐culture under perfusion with aAPC‐stimulated T‐cells alone, aAPC‐Adj–stimulated T‐cells, or aAPC‐Adj‐MSLN4–stimulated T‐cells, with or without the addition of FFX‐ATEZ. (c) T‐cell infiltration into PDAC Scr PDOs or PDAC MSLN KD PDOs under the same conditions as in (b) after 7 days of perfusion. The data presented in the graphs represent the mean ± SEM of results obtained from two to four tumor‐on‐a‐chip.^****^ *p* < 0.001 (Two‐way ANOVA followed by Dunnett's comparisons test).

Following the successful ex vivo stimulation of PDAC patient‐derived T‐cells using aAPCs, we next assessed the response of PDAC PDOs to either aAPC‐Adj‐MSLN4–stimulated T‐cells (referred to as Adj‐MSLN4) alone, or in combination with the chemotherapeutic agent FOLFIRINOX (FFX) and the PD‐L1 inhibitor Atezolizumab (ATEZ) (referred to as +FFX‐ATEZ), both of which are currently under evaluation in clinical trials for PDAC [49]. To this end, we introduced either wild‐type MSLN‐expressing PDAC PDOs (PDAC Scr) or MSLN‐knockdown PDAC PDOs (PDAC MSLN KD) into tumor‐on‐a‐chip chambers. Knockdown of MSLN was confirmed by PCR and flow cytometry (Figure S1). The organoids were then exposed to various conditions involving aAPC‐stimulated T‐cells alone, aAPC‐Adj–stimulated T‐cells, or aAPC‐Adj‐MSLN4–stimulated T‐cells, with or without the addition of immunotherapeutic and chemotherapeutic agents on the day after co‐culture (Figure 6a).

After seven days of continuous perfusion with PDAC patient‐derived T‐cells, we observed a modest, though not statistically significant, reduction in organoid volume in PDAC Scr samples treated with the combination of vaccine and FFX‐ATEZ. In contrast, organoid volume remained stable across all conditions in MSLN‐knockdown organoids (Figure 6b). This trend was in agreement with the pattern of T‐cell infiltration: PDAC Scr organoids treated with Adj‐MSLN4‐primed T‐cells, with or without additional FFX‐ATEZ therapy, showed increased T‐cell infiltration compared to those treated with Adj‐only stimulated T‐cells. Importantly, no increase in T‐cell infiltration was detected in the PDAC PDOs with reduced mesothelin expression (PDAC MSLN KD) (Figure 6c). Altogether, these findings support a specific response elicited by the MSLN‐based nanovaccine peptides, marked by enhanced infiltration of PDAC patient‐derived T‐cells in MSLN‐expressing organoids, underscoring the antigen‐specific nature of the immune activation. The results also confirm that PDO‐T‐cell co‐cultures can provide meaningful assessment of the specific immune response in more complex in vitro set‐ups, such as the tumor‐on‐a‐chip.

### 3.7 FOLFIRINOX Neoadjuvant Treatment Decreases the Frequency of Activated Adj‐MSLN4‐stimulated PDAC Patient‐Derived T‐Cells

**FIGURE 7:**
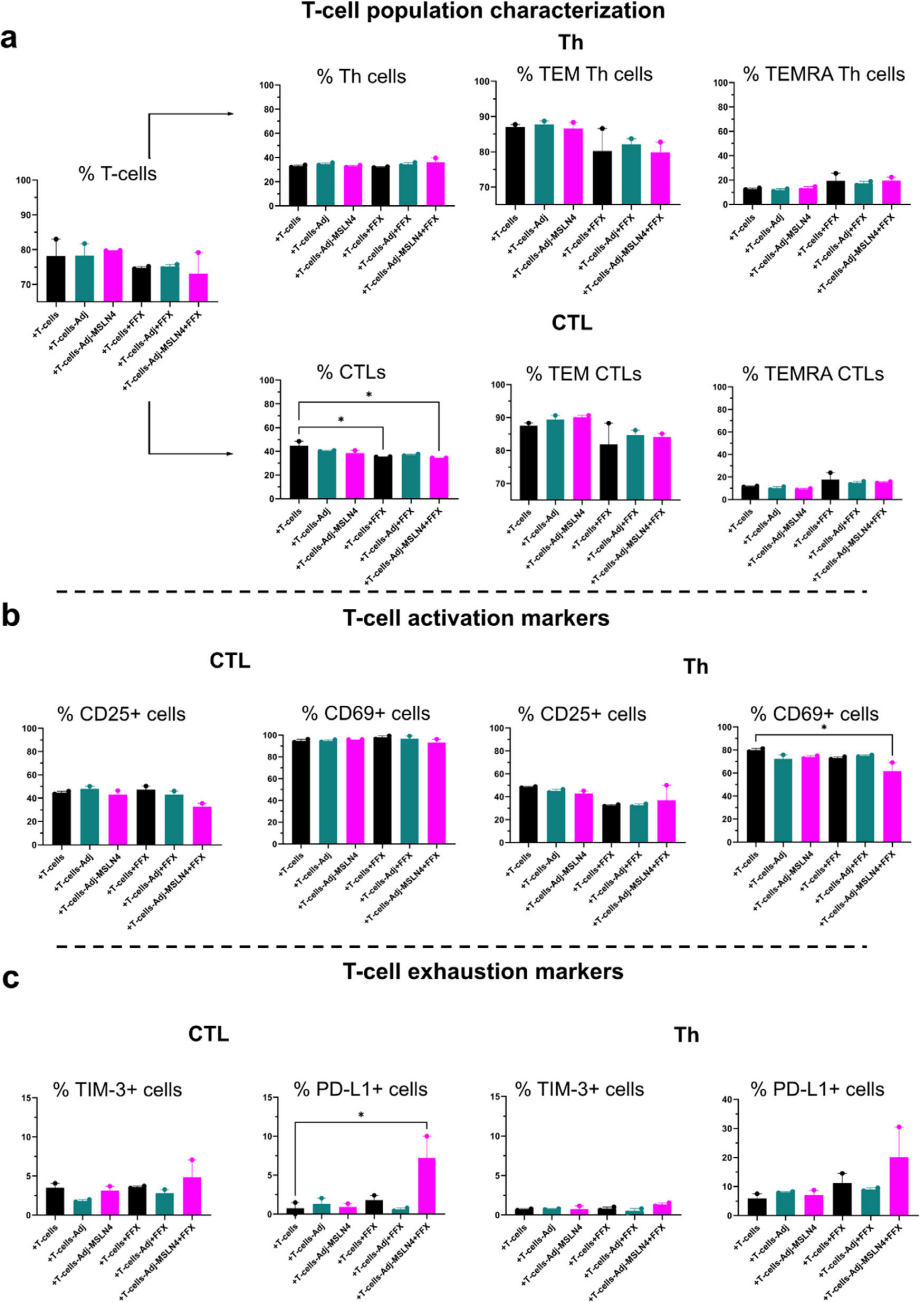
The addition of FOLFIRINOX to Adj‐MSLN4 stimulated PDAC T‐cells/PDO co‐cultures increases the frequency of exhausted CTLs and Th cells. (a) The T‐cell profile following co‐cultures of Adj‐MSLN4stimulated T‐cells with organoids, with or without the addition of FOLFIRINOX (FFX), was examined using flow cytometry. This analysis involved gating strategies to categorize general live T‐cells into CTLs and Th cells. Further stratification identified these cells as terminally differentiated effector memory (TEM) and terminally differentiated effector memory re‐expressing CD45RA (TEMRA) T‐cells. (b) The activation of Th cells and CTLs was assessed by employing antibodies against activation markers (CD25 and CD69) in PDAC patient‐derived T‐cells derived from the PDAC T‐cell/PDO co‐cultures. (c) Exhausted T‐cells were identified by the expression of TIM‐3 and/or PD‐L1 using flow cytometry. The data presented in the figures depict the mean ± SEM of samples obtained from two distinct biological PDAC T‐cell/PDO co‐cultures. ^*^ *p* < 0.05, ^**^ *p* < 0.01 (One‐way ANOVA followed by Dunnett's comparisons test).

To comprehend the impact of aAPC‐Adj‐MSLN4‐stimulated PDAC patient‐derived T‐cells when co‐cultured with PDAC organoids on the composition of immune cell populations, we conducted flow cytometry analysis. This analysis categorized the immune cells into CTLs and Th cells. We evaluated their activation and exhaustion profiles in co‐cultures of PDAC patient‐derived T‐cells and PDAC PDOs, both with and without the combination of chemotherapy and immunotherapy. We first examined the combination of these co‐cultures with FOLFIRINOX (FFX) alone (Figure 7). Notably, we observed a trend indicating a 5% reduction in the overall T‐cell population when FFX was added to the co‐cultures involving unstimulated, Adj‐, or Adj‐MSLN4‐stimulated PDAC patient‐derived T‐cells (Figure 7a). While there were no significant alterations in the total frequency of Th cells, we noticed a decrease of around 7% in effector memory (TEM) Th cells upon the addition of FFX to the co‐cultures compared to unstimulated T‐cell co‐cultures (+T‐cells). In terms of CTLs, a significant 5% decrease was observed when FFX was added to Adj‐MSLN4‐stimulated PDAC T‐cell/PDO co‐cultures (+T‐cells+Adj+MSLN4+FFX), compared to the unstimulated T‐cell/PDO co‐cultures (+T‐cells). A similar decreasing trend was also noted in the frequency of TEM CTLs. The number of terminally differentiated effector memory re‐expressing CD45RA (TEMRA) Th cells or TEMRA CTLs remained unaffected in the PDAC T‐cell/PDO co‐cultures. Concurrent with the general reduction in CTLs or TEM CTLs due to FFX addition in the co‐culture medium, CTLs exhibited lower numbers of the activation marker CD25, while CD69+ Th cells also decreased. Notably, only Adj+MSLN4+FFX stimulation of T‐cells (+T‐cells+Adj+MSLN4+FFX) led to a statistically significant decrease of the frequency of CD69+ Th cells by 20% compared to unstimulated T‐cell/PDO co‐cultures (Figure 7b, right panel). Furthermore, we evaluated the levels of PD‐L1 expressing cells, as an indirect exhaustion marker (ligand of the exhaustion marker PD1). Interestingly, under the same conditions, a notable 5% increase was observed in cells expressing PD‐L1, in both the CTL and Th cell populations (Figure 7c). Collectively, these findings indicate that combining Adj‐MSLN4‐stimulated T‐cells with FFX as an adjuvant chemotherapy leads to a higher number of exhausted CTLs and Th cells.

**FIGURE 8:**
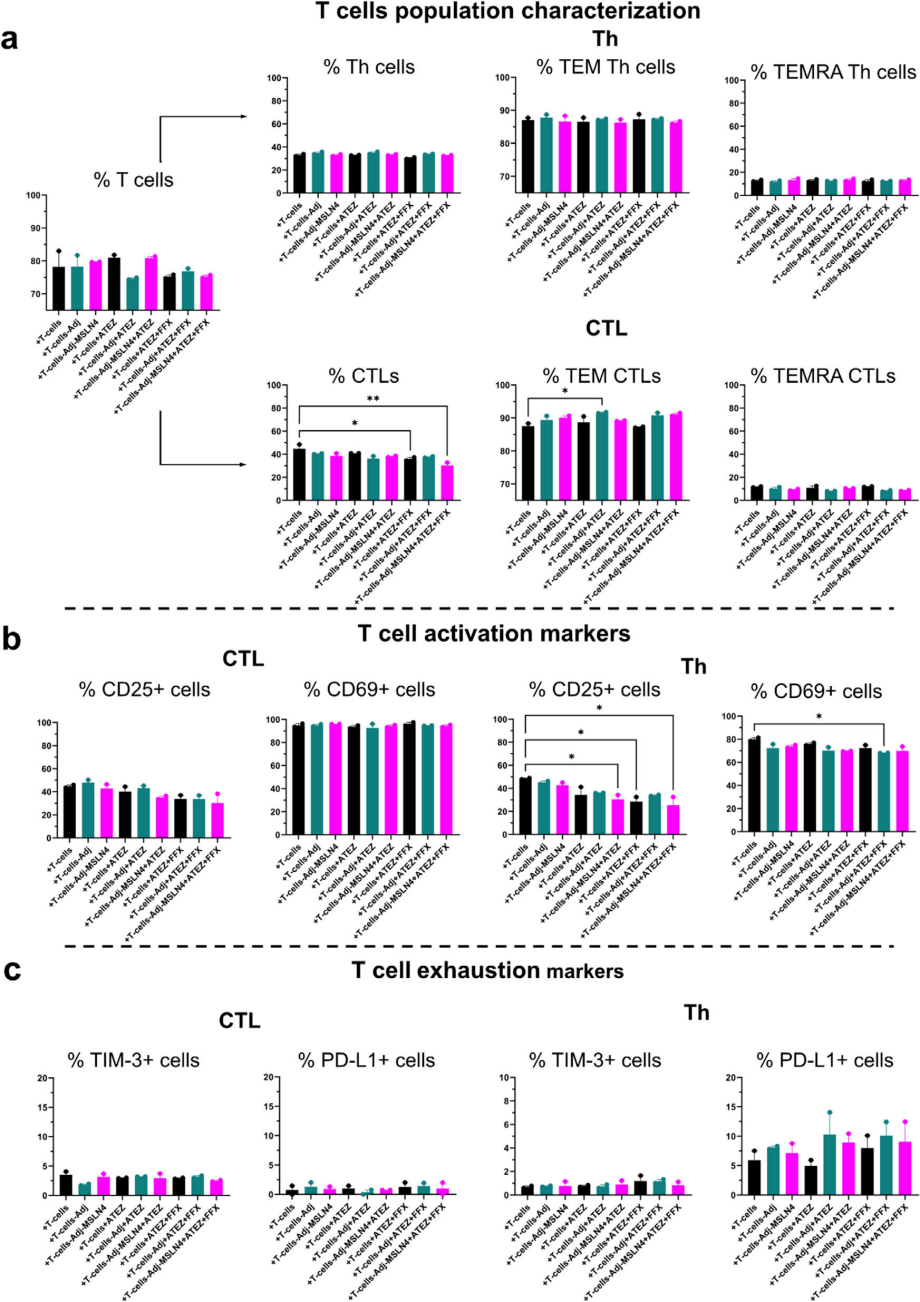
Atezolizumab alone or Atezolizumab combined with FOLFIRINOX treatment and Adj‐MSLN4‐stimulated PDAC T‐cell/PDO co‐cultures prevent exhaustion of PDAC patient‐derived T‐cells. (a) The T‐cell profile following co‐cultures of Adj‐MSLN4‐stimulated T‐cells with PDAC PDOs, treated with or without the immunotherapeutic Atezolizumab and combined with or without FOLFIRINOX (FFX), was examined using flow cytometry. This analysis involved gating strategies to separate CTL and Th cells from live T‐cells for quantification. Further stratification identified these cells as TEM and TEMRA T‐cells. (b) The activation status of CTLs and Th cells was assessed by employing antibodies against specific activation markers (CD25 and CD69) in T‐cells derived from the PDAC patient‐derived T‐cell/PDO co‐cultures. (c) T‐cell exhaustion was evaluated by the expression of TIM‐3 and/or PD‐L1. The data presented in the figures depict the mean ± SEM of samples obtained from two distinct biological PDAC T‐cell/PDO co‐cultures. ^*^ *p* < 0.05, ^**^ *p* < 0.01 (One‐way ANOVA followed by Dunnett's comparisons test).

T‐cell phenotyping was also conducted following co‐cultures of PDOs with T‐cells‐Adj‐ or Adj‐MSLN4‐stimulated T‐cells together with the PD‐L1‐inhibitor Atezolizumab (ATEZ) or Atezolizumab in combination with FOLFIRINOX (ATEZ+FFX). Only a marginal decrease of around 5% was observed in the total T‐cell count when ATEZ+FFX was included in the medium (Figure 8a). In the case of Th cells, there was no discernible effect from the various combinations in the co‐cultures. However, a significant decrease of 15% in CTL frequency was noted in Adj‐MSLN4‐stimulated T‐cell/PDO cultures with added ATEZ+FFX treatment (+T‐cells‐Adj‐MSLN4+ATEZ+FFX) compared to unstimulated T‐cell/PDO co‐cultures (+T‐cells). However, there was rather a trend toward increased frequency of TEM CTLs in all conditions, with statistical significance only observed in T‐cell‐Adj+ATEZ‐stimulated PDAC T‐cell/PDO co‐cultures (+T‐cells‐Adj+ATEZ). The frequencies of TEMRA CTLs or TEMRA Th population remained unaltered. A 10% and 20% reduction in CD25 positive cells was seen in both CTL and Th cell populations, respectively, from co‐cultures with ATEZ+FFX or Adj‐MSLN4‐stimulated T‐cell/PDO co‐cultures combined with ATEZ+FFX (+T‐cells‐Adj‐MSLN4‐ATEZ+FFX). A significant decrease of 10% in the number of CD69+ Th cells in the Adj‐stimulated T‐cell/PDO co‐cultures in response to ATEZ+FFX (Figure 8b) was also noticed (+T‐cells‐Adj+ATEZ+FFX). Despite the decrease in activation markers, neither TIM‐3 nor PD‐L1 positive cells showed any alteration under the various conditions tested (Figure 8c). In summary, despite a minor decrease in activated T‐cells, the treatment of Adj‐MSLN4 with ATEZ+FFX prevented the exhaustion of the T‐cells, identified by an unchanged number of PD‐L1+ and TIM‐3+ cells. This is in contrast to +T‐cell‐Adj‐MSLN4+FFX treatment, which increased the number of PD‐L1+ and TIM‐3+ T‐cells (Figure 7c), suggesting that the addition of ATEZ regulated this exhausted T‐cell population. Thus, the triple combination +T‐cells‐Adj‐MSLN4+ATEZ+FFX keeps the functional state of the T‐cells throughout the co‐cultures. On the other hand, neither the combination with the chemotherapeutic drug nor the immunotherapeutic therapies promoted the activation of the PDAC CTLs and Th cells throughout the co‐cultures.

### 3.8 Combination of Atezolizumab and FOLFIRINOX Enhances Adj‐MSLN4 Anti‐Tumoral Efficacy in PDAC PDOs

**FIGURE 9:**
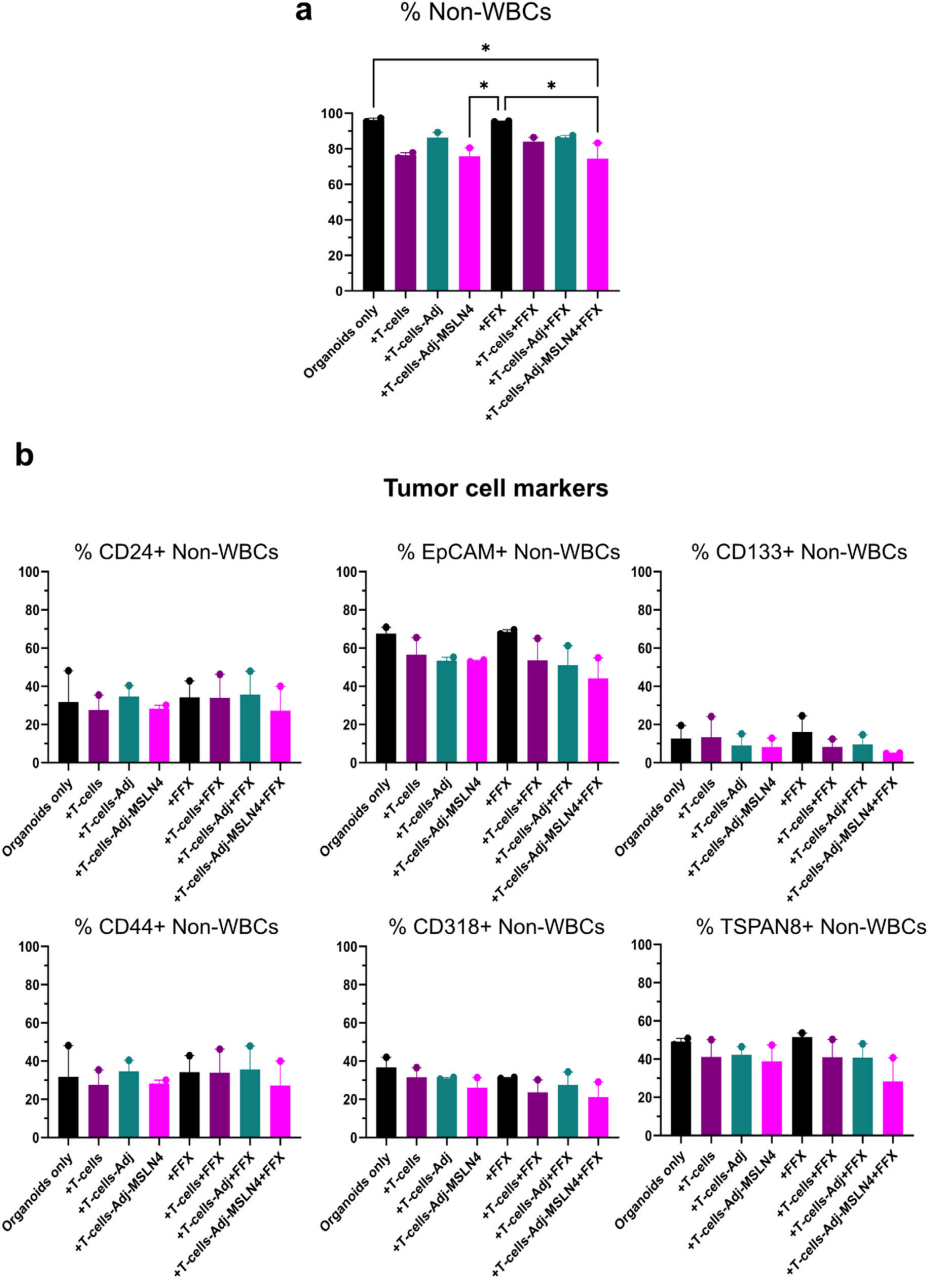
Reduced frequency of total tumor cells, CSCs and PDAC‐associated marker‐positive cells in PDAC T‐cell/PDO co‐cultures under +T‐cells‐Adj‐MSLN4+FFX condition. (a) Following 4 days of co‐cultures with either unstimulated, Adj‐, or Adj‐MSLN4‐stimulated PDAC T‐cells/PDOs with or without FOLFIRINOX (+FFX), flow cytometry was utilized to quantify the total number of live tumor cells (% Non‐White Blood Cells (WBC)) after each treatment. The frequency of Non‐WBCs was compared to untreated PDAC PDOs (PDOs only). (b) Flow cytometry was also employed to quantify the frequency of CSCs (CD24, EpCAM, CD133, and CD44 expression positive) and Non‐WBCs expressing PDAC tumor‐associated markers (CD318, TSPAN8). The graphs present data as the mean ± SEM derived from samples obtained from two distinct PDAC T‐cell/PDO co‐cultures. ^*^ *p* < 0.05 (One‐way ANOVA followed by Holm–Šídák's multiple comparisons test).

To elucidate the specific impact unstimulated (+T‐cells), Adj‐ (+T‐cells‐Adj) or Adj‐MSLN4‐stimulated (+T‐cells‐Adj‐MSLN4) PDAC T‐cells have on distinct populations of tumor cells in T‐cell/PDO co‐cultures alone or in the presence of chemo‐ or immunotherapy, we performed flow cytometry analysis to quantify both the total number of tumor cells (% Non‐WBCs) and the prevalence of cancer stem cells (CSCs) by applying antibodies against specific CSCs markers (CD24, CD133, EpCAM, CD44) [50]. Additionally, we assessed potential targets for PDAC immunotherapy, including TSPAN8 and CD318 [51], after 84 h of co‐culture. A reduction of approximately 20% in the frequency of tumor cells (% Non‐WBCs) was observed in co‐cultures containing +T‐cell‐Adj‐MSLN4 and +T‐cell‐Adj‐MSLN4+FFX (Figure 9a). Similarly, conditions involving unstimulated T‐cells (+T‐cells) displayed a capacity to reduce an equivalent amount of tumor cells (Figure 9a). Notably, although not statistically significant, a tendency toward decreased CSC numbers (markers CD24, EpCAM, and CD133) was observed when PDAC PDOs were co‐cultured with Adj‐MSLN4‐stimulated T‐cells. This trend was also observed in the numbers of TSPAN8 and CD318 positive (PDAC‐associated markers) tumor cells. Conversely, a slight increase in cells expressing CD44, another PDAC tumor‐associated marker, was noticed with all treatment combinations compared to untreated controls. This increase might suggest a potential resistance mechanism, as CD44 is associated with PDAC chemoresistance [52]. The diminished effect on CSCs and PDAC cells appeared more pronounced in Adj‐MSLN4‐stimulated T‐cell/PDO co‐cultures in the presence of FOLFIRINOX (+T‐cells‐Adj‐MSLN4+FFX) (Figure 9b).

**FIGURE 10:**
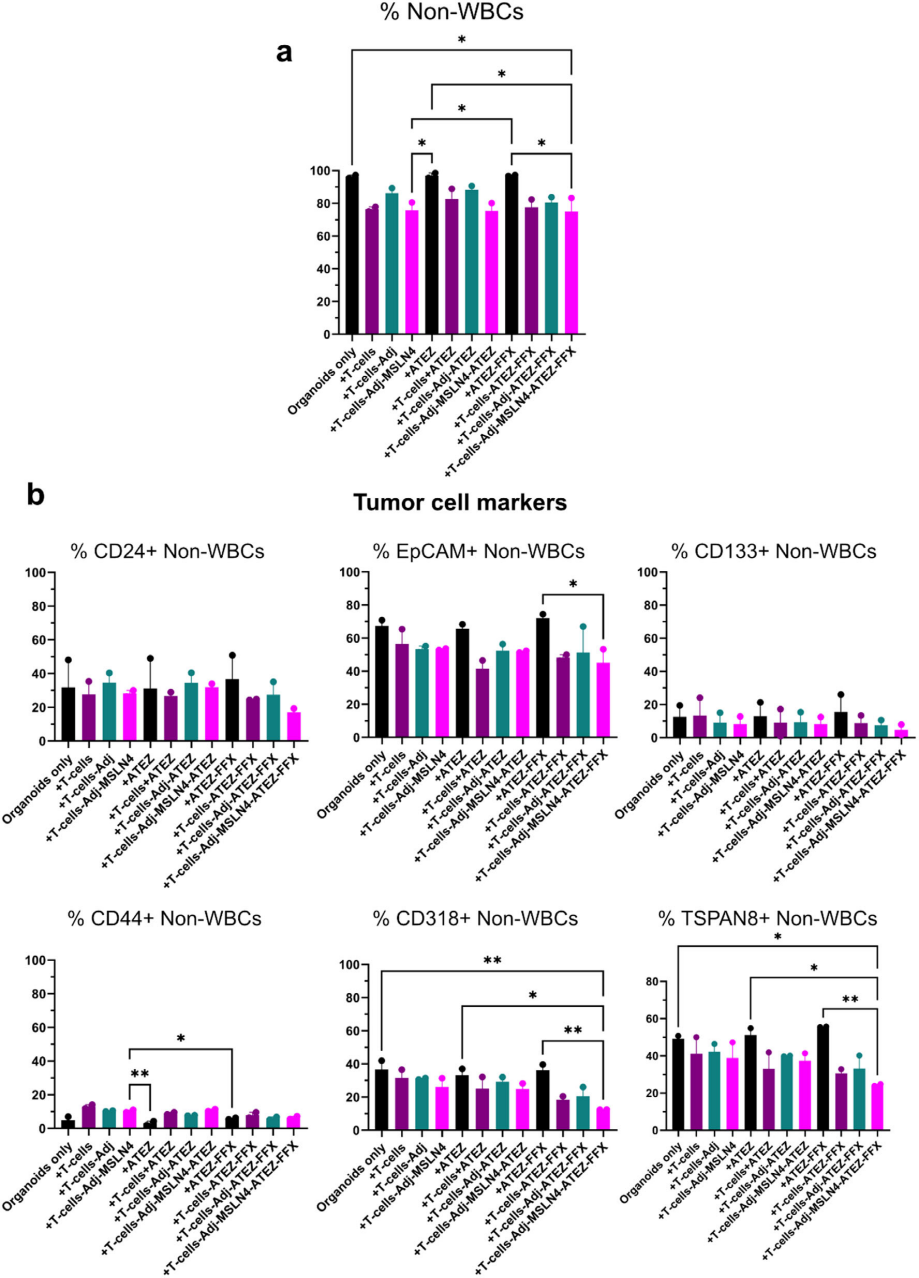
Adj‐MSLN4 combined with Atezolizumab and FOLFIRINOX treatment of PDAC T‐cell/PDO co‐cultures results in a reduction of PDAC‐tumor‐marker‐positive cells and in the inhibition of the increase of resistance‐associated CD44+ cells. (a) Following four days of PDAC T‐cell/PDO co‐cultures involving unstimulated T‐cells, Adj‐, or Adj‐MSLN4‐stimulated PDAC patient‐derived T‐cells/organoids with or without a combination of immunotherapeutics (+ATEZ or +ATEZ‐FFX), flow cytometry was utilized to quantify the total number of live tumor cells (% Non‐WBCs) after each treatment. The total number of live Non‐WBCs in organoids was compared to untreated PDAC PDOs (Organoids only). (b) Flow cytometry was employed to quantify the number of CSCs (CD24, EpCAM, CD133, CD44) and PDAC tumor‐associated marker‐expressing cells (CD318, TSPAN8). The graphs present data as the mean ± SEM derived from samples obtained from two distinct human PDAC T‐cell/PDO co‐cultures. ^*^ *p* < 0.05, ^**^ *p* < 0.01, ^***^ *p* < 0.001 (One‐way ANOVA followed by Holm–Šídák's multiple comparisons test).

The addition of Atezolizumab (+T‐cells‐Adj‐MSLN4+ATEZ) alone or combined with FOLFIRINOX (+T‐cells‐Adj‐MSLN4+ATEZ+FFX) did not alter the approximately 25% decrease in the number of total tumor cells (% Non‐WBCs) that we observed with unstimulated (+T‐cells) or Adj‐MSLN4‐stimulated (+T‐cells‐Adj‐MSLN4) T‐cells (Figure 10a). Concerning CSCs, the highest decrease in CD24/EpCAM/CD133‐positive cells was seen with the addition of both Atezolizumab and FOLFIRINOX (+T‐cells‐Adj‐MSLN4+ATEZ) (Figure 10b). Atezolizumab alone and in combination with FFX did not lead to a reduction in CSCs (Figure 10b, upper panel). Only the combination of ATEZ and FFX with MSLN4‐stimulated T‐cells (+T‐cells‐Adj‐MSLN4+ATEZ+FFX) significantly decreased the number of EpCAM+ CSCs. Regarding PDAC tumor‐associated markers, a significant decrease of 30% in CD318 and 25% in TSPAN8 expressing Non‐WBCs was observed in the co‐cultures of Adj‐MSLN4‐stimulated PDAC T‐cells/PDOs with ATEZ‐FFX treatment (+T‐cells‐Adj‐MSLN4+ATEZ+FFX) compared to untreated PDAC organoids (Organoids only) and ATEZ or ATEZ+FFX alone. Interestingly, a significant increase of approximately 5% was detected in CD44+ Non‐WBC from Adj‐MSLN4‐stimulated T‐cell/organoids PDO co‐cultures (+T‐cells‐Adj‐MSLN4) and in their respective controls (+T‐cells and +T‐cells‐Adj), as well as with the addition of ATEZ (+T‐cells‐Adj‐MSLN4+ATEZ). However, this effect was not observed in the Adj‐MSLN4‐stimulated co‐cultures in response to ATEZ‐FFX treatment, suggesting that ATEZ‐FFX counteracts a potential resistance mechanism of PDAC organoids through control of the CD44+ population.

In summary, by reducing the total number of tumor cells, in particular CSCs and non‐WBCs that express the PDAC‐associated tumor marker CD318 and TSPAN8, Adj‐MSLN4 treatment demonstrates its potential to induce the killing of PDAC PDOs. Moreover, the addition of Atezolizumab and FOLFIRINOX to the MSLN4‐stimulated T‐cells not only potentiated the antitumor effect but also inhibited the proliferation of resistant CD44+ cells. The pipeline once again demonstrates that even small differences in immune response can be detected in PDAC T‐cell/PDO co‐cultures, supporting more accurate therapeutic choices.

## 4 Discussion

In this study, we devised a strategic in vitro workflow of multiple steps applying advanced human immune cell‐organoid co‐cultures in combination with different therapy strategies that allow a thorough analysis of the specificity and effectivity of a nanovaccine formulation. Starting with the characterization of the T‐cell activation in dependence of the formulation components and continuing with the expansion and profiling of reactive T‐cells by employing aAPCs, we ensured an in‐depth quantification of T‐cell immune response to the nanovaccine. We then used this information to set up sandwich‐based co‐cultures of PDAC patient‐derived T‐cells and HLA‐matched PDAC PDOs to evaluate nanovaccine efficiency and specificity, including advanced in vitro models such as tumor‐on‐a‐chip. While organoid–immune cell co‐cultures have been previously described [53], the novelty of this study lies in the establishment of this stepwise workflow. This integrated system enables, for the first time, the in vitro evaluation of cancer vaccines either as monotherapies or in combination with other therapeutic modalities, providing a patient‐relevant model that offers a powerful tool for personalized immunotherapy preclinical testing.

As a use case for this workflow, we explored a mesothelin‐based nanovaccine formulation (Mesovac), a peptide which has been repeatedly investigated as a promising PDAC target [7]. Despite the unsatisfactory clinical translation of the mesothelin target using different approaches (ADCs, CAR‐T‐cells, vaccines), nanovaccination and combinatorial approaches, as we use them here, have not been attempted before. Mesothelin therefore, served as a suitable target to evaluate these alternatives in a new in vitro setting. Consistent with earlier findings, our IHC analysis revealed a varied MSLN expression in PDAC PDOs. Hagerty et al. [54] found an association between mesothelin expression levels with transcriptomics pathways connected with tumor aggressiveness, with a correlation between high MSLN levels and enhanced proliferation, decreased immune response, and sensitivity to chemotherapy. Different levels of MSLN expression, as we found in PDAC PDOs, show the importance of using personalized pre‐clinical models to identify with accuracy the potency of mesothelin targeting strategies.

We stratified reactive T‐cells by flow cytometry from unfractionated PBMCs from healthy donors following in vitro stimulation with different MSLN peptide sequences or with Mesovac and its respective PLGA‐encapsulated controls. Although the percentage of T‐cells, neither Th cells or CTLs, did not increase with any of the individual components, we found a notable trend toward an increase of intracellular IFN‐γ levels in CTLs and Th cells, particularly when stimulated with MSLN3 and MSLN4 sequences. Both peptide‐sequences represent synthetic long‐peptide sequences (SLPs). This characteristic accounts for their higher immunogenicity levels in comparison to MSLN2, along with their capacity to be taken up and to be processed by both HLA class I and II molecules, leading to the induction of strong and long‐lasting antitumor responses by the induction of long‐term T‐cell memory [16]. We noted a high variability in the stimulation reflected by a large standard error, suggesting a possible variability in the response of individual PBMC donors. Moreover, the detection of peptide‐activated T‐cells in vitro is inherently difficult as the percentage in activated cells is very low and the sensitivity of T‐cells toward stimulation is notoriously variable [55]. Thus, our findings of a consistent trend of INF‐γ‐positive T‐cells in response to MSLN3 and MSLN4 as much or higher as the adjuvants indicated that Mesothelin peptides could indeed activate T‐cells. The MSLN4‐loaded aAPC technology used to stimulate and expand reactive T‐cells revealed a successful increase count of IFN‐γ positive cells as well as higher levels of IFN‐γ production over time after stimulation with aAPCs coupled to the full components in the Mesovac formulation. This effective expansion of T‐cells with peptide‐coupled aAPCs as opposed to the weak activation of T‐cells, that we saw in the in vitro stimulation of PBMCs of healthy donors, highlights the importance of the antigen presentation for T‐cell activation. The mesothelin peptide evidently did produce a significant immune response when APCs were abundantly present in our ex vivo approach.

Ex vivo expanded PDAC T‐cells show increased Th and CTL populations, with Th cells displaying a TEMRA‐like, highly functional profile. This might be due to the chronic antigen exposure that promotes a terminally differentiated profile in cytotoxic CD4+ T‐cells [56]. These cells also exhibited higher CD25 and lower CD39 expression, indicating an activated, non‐exhausted state. A similar profile was described by Hirschhorn–Cymerman et al. [57], in which OX40 stimulation led to a TEMRA Th cell profile with memory/low exhaustion markers in Trp1 cells, responsible for controlling advanced tumors. In summary, the observed profile indicates a robust and activated state of Th cells, which is generally favorable for an effective immune response against PDAC or other relevant stimuli. This heightened responsiveness may contribute to enhanced immune surveillance and potential effectiveness in combating the targeted condition. Future work will require testing more T‐cells from tumors of different PDAC patients. A different tendency was observed using T‐cells from PBMCs from healthy donors, in which we observed a modest increase in TEM CTLs with MSNL4 stimulation. This divergence of the results obtained with T‐cells from healthy donors vs. T‐cells from PDAC patients, shows the importance of using tumor‐derived T‐cells as they better represent the phenotype of immune cells in a PDAC microenvironment.

After expanding reactive PDAC T‐cells to enable direct contact with PDAC PDOs in a stable position, we optimized a Matrigel sandwich protocol [20, 21], in which we trapped stimulated PDAC T‐cells with PDAC PDOs. Following the 3 days of co‐culture, we characterized the general immune cell and tumor cell populations by flow cytometry. The introduction of FOLFIRINOX resulted in a reduction of CTLs and TEM cells, particularly evident in Adj‐MSLN4‐stimulated PDAC T‐cell/PDO co‐cultures. This observation suggests a potential impact of FOLFIRINOX on T‐cell dynamics within the immune cell population. Moreover, the addition of FOLFIRINOX led to a decrease in activation marker expressing cells and an increase in PD‐L1 expressing cells, particularly in CTLs and Th cells stimulated with T‐cell‐Adj‐MSLN4. These observations imply a shift toward immune cell exhaustion, particularly in Adj‐MSLN4 and FOLFIRINOX combined treatment, indicating a possible compromise in T‐cell functionality and response efficacy. The combination of Atezolizumab and FOLFIRINOX led to a slight reduction in total T‐cell and CTL counts but preserved T‐cell function, as indicated by stable exhaustion marker expression, highlighting Atezolizumab's potential role in maintaining T‐cell activity during adjuvant treatment with Adj‐MSLN4. This might indicate the importance of the PD‐L1 inhibitor Atezolizumab as a treatment strategy through the maintenance of T‐cell functionality.

In an advanced model of co‐culture in tumor‐on‐a‐chip, we were able to demonstrate the specificity of Adj‐MSLN4‐T‐cell response by the higher infiltration of the reactive T‐cells only into highly MSLN‐expressing PDAC PDOs. Moreover, MSLN‐expressing PDAC PDO volumes were generally reduced with the triple combination Adj‐MSLN4/Atezolizumab/FOLFIRINOX when compared with MSLN low expressing PDOs, demonstrating an antitumor effect. These results also demonstrate that our co‐culture models are able to detect differences in therapy in dependence on antigen expression level, suggesting that in patients lacking expression of the target protein, this platform could predict limited vaccine efficacy. This highlights the translational potential of our system in the context of personalized medicine, enabling pre‐treatment assessment of vaccine responses. Nevertheless, this in vitro approach has its limitations too. The immunosuppressive milieu of PDAC is highly complex, driven by interactions among various cell populations, including tumor‐associated macrophages, regulatory T‐cells, myeloid‐derived suppressor cells, and fibroblasts [58]. To expand the complexity of the co‐cultures, such additional components should be explored in the future.

Last but not least, we assessed the impact of various treatment combinations on PDAC organoid tumor cells, focusing on cancer stem cell (CSC) markers [59] and tumor‐associated antigens [51]. T‐cell‐Adj‐MSLN4 combined with FOLFIRINOX reduced overall tumor cell numbers and showed a clear decrease in CSCs expressing CD24, EpCAM, and CD133. Adding Atezolizumab further diminished CSCs and significantly reduced CD318 and TSPAN8‐expressing cells. Most importantly, it maintained the levels of CD44 expressing tumor cells, which are involved in pancreatic cancer plasticity, invasiveness, and resistance to chemotherapeutic treatment [52, 60]. Overall, the triple combination led to a reduction in aggressive tumor subpopulations previously linked to poor prognosis in PDAC [61]. Further experiments are necessary to evaluate MSLN co‐expression with these CSC markers and the probability that the MSLN vaccine reduces CSC self‐renewal capacity.

In summary, this study establishes a robust and versatile ex vivo platform that integrates patient‐derived T‐cells, PDAC organoids, and immunomodulatory treatments to evaluate the specificity, efficacy, and mechanistic impact of an MSLN‐based nanovaccine. The workflow enables detailed characterization of antigen‐specific T‐cell responses, including infiltration into MSLN‐expressing organoids, cytokine production, and cytotoxic activity against tumor‐initiating subpopulations. Notably, the combinatorial approach combining Adj‐MSLN4, FOLFIRINOX, and Atezolizumab treatment with the nanovaccine approach not only sustained T‐cell activation but also reduced tumor cell populations associated with therapeutic resistance. For clinical relevance, assessment of the combination treatment MSLN‐FFX‐ATEZ should include formal interaction assays to better understand the additive or synergistic function of each component. Moreover, despite the encouraging therapeutic potential of the combined therapy, its efficacy should be validated in a larger cohort of PDAC PDOs and corresponding PDAC‐derived T‐cells. Despite donor‐to‐donor variability and low sample size, the system effectively captures individual immune reactivity, highlighting its translational potential for tailoring and optimizing therapeutic strategies. Altogether, this pipeline represents a powerful tool for dissecting complex immunotherapeutic responses in vitro and holds significant promise for the future development of personalized treatment regimens in pancreatic cancer.

## 5 Conclusion

Although animal models represent a fundamental model for cancer vaccine preclinical testing, it continue to poorly predict the effect in humans. As organoids remain the genetic features of patient‐derived tumor tissue, we represent the first attempt to evaluate a cancer vaccine effect in organoid/immune cell co‐cultures. With a multi‐approached pipeline, we used a peptide sequence of mesothelin, as a known tumor‐associated antigen, in the cancer nanovaccine formulation, named Mesovac. Testing alone or in combination with other PDAC chemotherapeutics or immunotherapeutics options, we observed a possible synergy of the Mesovac components, Atezolizumab, and FOLFIRINOX triple treatment in targeting mesothelin expressing PDAC PDOs.

## Author Contributions

N.F. conceived and designed the experiments, acquired and analyzed the data, and wrote the paper. A.K. developed OrganoIDNet, and contributed toward analyzing the data, D.A. performed the FACS analysis. O.L. and F.Albericio synthesized the MSLN peptides. S.S. and L.C. manufactured Mesovac nanovaccine and respective controls. A.H. evaluated the MSLN mRNA levels from PDAC PDOs. M.A.M., F.R.G., and F.Alves contributed to conceptualizing the experiments, supervision, and writing the manuscript. T.L. provided the HLA‐matched PBMCs, and P.S. provided the patient‐derived PDAC organoids. P.B. provided the PDAC T‐cell. L.C. produced the tumor‐on‐a‐chip. N.F., A.R., and J.R. conducted the tumor‐on‐a‐chip co‐culture. A.R., N.F., N.A., and P.L. designed the tumor‐on‐a‐chip co‐culture experiment. All authors discussed the data and reviewed the manuscript.

## Funding

This project has received funding from the European Union's Horizon 2020 research and innovation program under the Marie Skłodowska–Curie grant agreement No. 861190 (PAVE), and by the Ministry for Science and Culture of Lower Saxony as part of the project “Agile, bio‐inspired architectures” (ABA). This work was supported by the Scientific Service Facility Cell Sorting at the University Medical Center Göttingen (Germany) through the DFG project number (DFG project number 442249343, BD LSRFortessa X‐20, Becton Dickinson).

## Conflicts of Interest

The authors declare no conflicts of interest.

## Supporting information




**Supporting File**: advs73516‐sup‐0001‐SuppMat.docx.

## Data Availability

The data that support the findings of this study are available from the corresponding author upon reasonable request.
