# Supplementary material for: Advanced Human Immune Cell‐Organoid Co‐Cultures for Functional Testing of Cancer Nanovaccines
Source: Adv Sci (Weinh). 2025 Dec 23;13(10):e15199. doi: 10.1002/advs.202515199 (PMC12915205; doi:10.1002/advs.202515199)
Supplement: Supplementary file 1 — Supporting File: advs73516‐sup‐0001‐SuppMat.docx. [file ADVS-13-e15199-s001.docx]

**Supplementary material and methods**

**Preparation of organoid medium**

**Digestion medium**. Per 100 ml: 12 mg Collagenase type I (Sigma), 12 mg Dispase II (Sigma), and 1 ml of 10% FCS (Gibco) were added to 99 ml of DMEM (Gibco).

**Organoid passaging medium.** Per 500 ml: 5 ml 100x Glutamax (Gibco), 5 ml 1 M HEPES (Gibco) and 1% P/S were added to Advanced DMEM/F12 medium (AdDMEM/F12, Gibco).

**Human organoid growth medium (HOGM).** Per 50 ml of HOGM: 25 μl A83-01 (1 mM, Tocris), 50 μl Human Epidermal Growth Factor (hEGF; 500 μg/ml, Invitrogen), 50 μl human Fibroblast Growth Factor-10 (hFGF-10; 100 mg/ml, Peprotech), 50 μl Gastrin I (100 μM, Sigma), 125 μl N-acetylcysteine (500 mM, Sigma), 500 μl Nicotinamide (1 M, Sigma), 1 ml B-27 supplement (50x, Gibco), 100 μl Primocin (50 mg/ml, InvivoGen), 25 ml of Wnt3a-, 5 ml R-spondin and 50 μl of Noggin-conditioned media were diluted in 19 ml of organoid splitting medium (1x Glutamax (Gibco), 1x HEPES (Gibco), 1 ml 1x Primocin (InvivoGen), 30% Bovine Serum Albumin (BSA, Sigma, diluted in AdDMEM/F12 (Gibco). For the initial seeding, splitting, or thawing, 1:1000 Rho Kinase Inhibitor (Sigma) was added to the organoid medium.

**Wnt3a-, R-Spondin- and Noggin-conditioned media.** For the preparation of Wnt3a-conditioned media, the L-Wnt3A cell line (ATCC® CRL-2647™) was acquired from ATCC and cultured in adherence to the guidelines provided by the manufacturer. A detailed protocol can be found in the work of Wilson *et al*^[1]^ To generate R-spondin- and Noggin-conditioned media, 293T-HA-Rspol-Fc (obtained from Calvin Kuo's group at Stanford University) and HEK293-mNoggin-Fc (sourced from AG Florian Greten and AG Herner Farin at Georg-Speyer-Haus Frankfurt) cell lines were utilized. The preparation followed the protocol outlined by Klemke *et al.*^[2]^ Specifically, 293T-HA-Rspol-Fc or HEK293-mNoggin-Fc cells were thawed and seeded in 175 cm^2^ flasks with growth medium (comprising 500 ml DMEM, Gibco, 60 ml FCS, and 5 ml penicillin/streptomycin (P/S) supplemented with 300 μg/ml Zeocin (Invitrogen) or 500 μg/ml Geneticin (Gibco)). Upon reaching confluency, cells were expanded into eight (for 293T-HA-Rspol-Fc cells) or six (for HEK293-mNoggin-Fc cells) 175 cm^2^ flasks using antibiotic-free medium. To preserve the cells, one flask from each condition was aliquoted and supplemented with Zeocin or Geneticin before freezing. Subsequently, upon reaching confluency, the antibiotic-free medium was replaced with 50 ml of conditioning medium (AdDMEM/F12 medium supplemented with 1x Glutamax, 1M HEPES (Invitrogen), and 1% P/S). After one week of culturing, the conditioned medium was collected, centrifuged (500xg, 4°C, 5 min), pooled from all the flask supernatants, and then filter-sterilized using a 0.22 µm filter. The resulting R-spondin- or Noggin-conditioned media were collected into 15 ml conical tubes (5 ml per tube) and stored at -20°C.

**Table S1. List of antibodies (Biolegend) used for validation of *in vitro* human PBMCs activation.**

| **Target** | **Conjugate** | **Catalog number** |
| --- | --- | --- |
| CD3 | FITC | 344803 |
| CD8 | APC | 301049 |
| CD4 | BV | 300554 |
| IFN-γ | PE | 506507 |
| TNF-ɑ | PE/CY7 | 502930 |

**Table S2. List of antibodies (Miltenyi Biotec) used for PDAC PDO and T cell characterization by flow cytometry.**

| **Target** | **Conjugate** | **Catalog number** |
| --- | --- | --- |
| EpCAM | VioGreen | 130-111-005 |
| TSPAN8 | FITC | 130-106-814 |
| CD133 | PE | 130-113-108 |
| CD45 | PerCP-Vio700 | 130-110-636 |
| CD44 | PE-Vio770 | 130-113-343 |
| CD318 | APC | 130-101-249 |
| CD24 | APC-Vio770 | 130-112-659 |
| CD45RA | VioBlue | 130-120-033 |
| CD4 | VioBright FITC | 130-113-220 |
| CD3 | PE | 130-113-139 |
| CCR7 | APC | 130-120-460 |
| CD8 | APC-Vio770 | 130-110-681 |
| CD56 | VioBright B515 | 130-114-552 |
| CD45 | VioGreen | 130-110-638 |
| TIM-3 | PE-Vio770 | 130-121-334 |
| EpCAM | PerCP-Vio700 | 130-111-003 |
| CD3 | VioGreen | 130-113-142 |
| CD25 | VioBright B515 | 130-115-536 |
| CD69 | PE-Vio770 | 130-112-615 |
| PD-L1 | PE | 130-122-809 |

**Table S3. Overview of the mutational profile of human PDAC organoids used in this study.** WT: Wild Type, NA: Not Applicable, VUS: Variant of uncertain significance


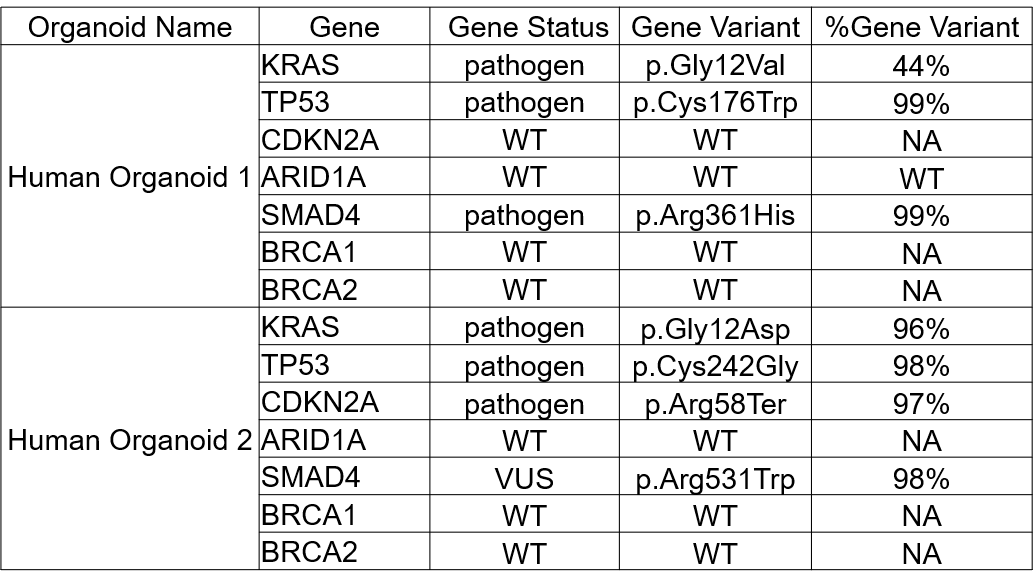


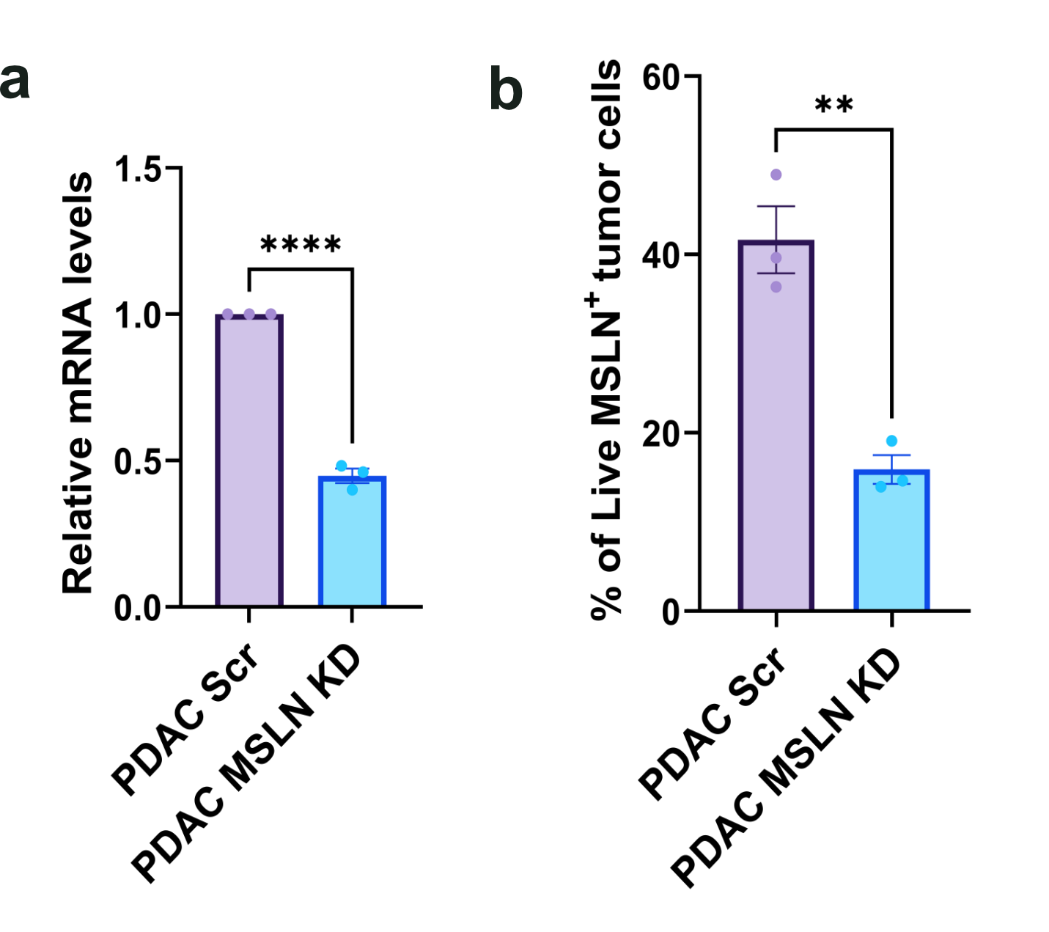


**Figure S1. Confirmation of MSLN knowdown in PDAC PDOs. Graphs showing real-time qPCR analysis mRNA levels** (a) and flow cytometry analysis (b) of PDAC PDOs transfected with a vector containing scrambled shRNA (PDAC Scr) or with Mesothelin knockdown vector (PDAC MSLN KD) for 72 h. Unpaired Student’s two-tailed t-test, **p < 0.01, ****p < 0.0001 of 3 technical replicates. Note that transfection with Mesothelin knockdown vector led to a reduction of about 60% in mRNA and protein MSLN levels.


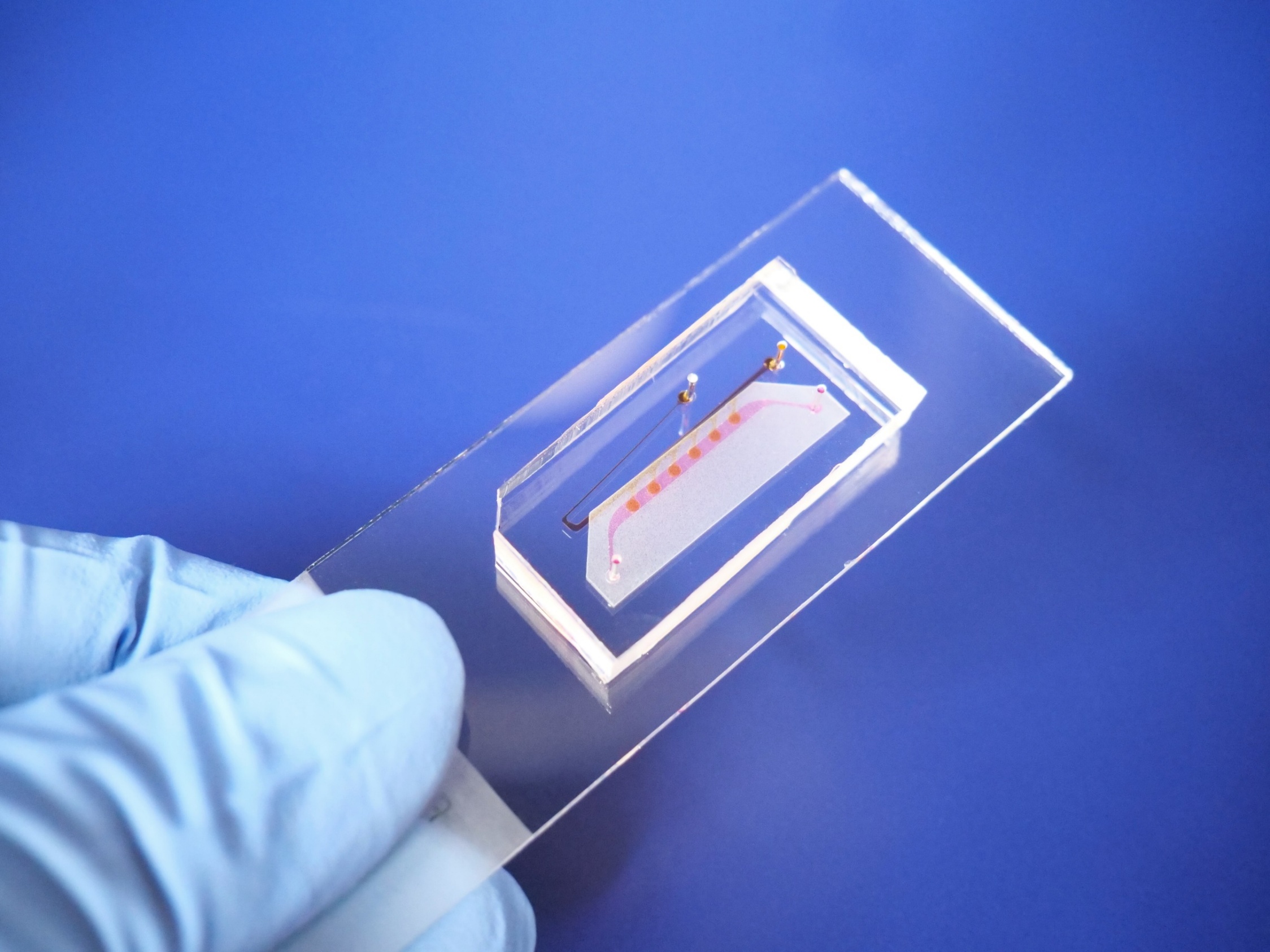


**Figure S2. Schematic of the tumor-on-chip model.** Tumor organoids embedded in a dextran-based hydrogel were cultured in 6 cylindrical tumor chambers per chip. The tumor chambers (yellow) lie beneath the medium channel (purple), separated by a PET membrane. Control or MSLN-stimulated T cells were perfused through the medium channel and migrated into the tumor chambers.

**
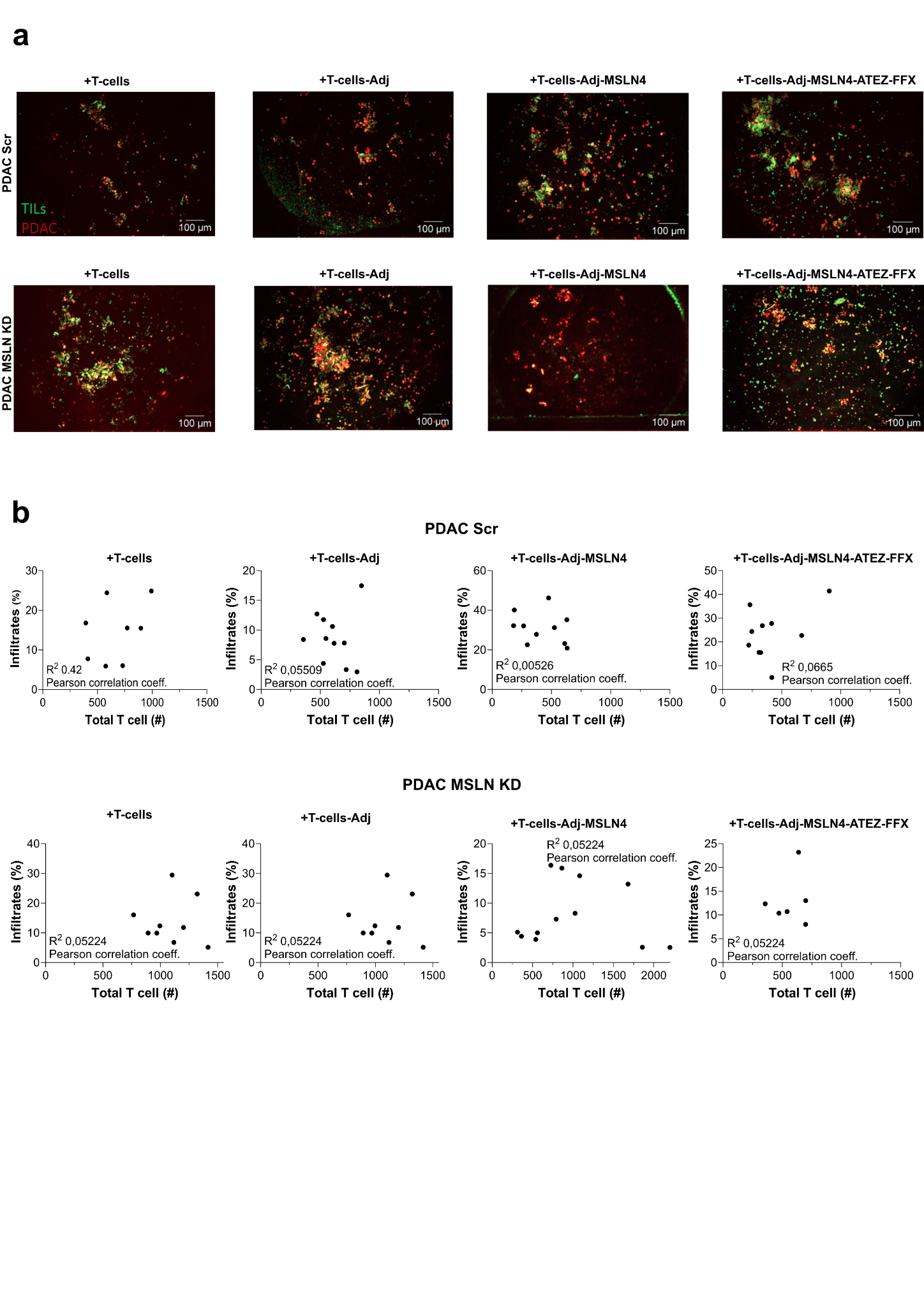
**

**Figure S3. Quality control of Tumor-on-a-chip design.** (a) Representative confocal images after 7 days from the co-cultures of Scramble (Scr) or Mesothelin Knockdown (KD) PDAC organoids identified by mCherry expression (Red) and PDAC Tumor infiltrating lymphocytes (TILs) identified by the cysteine-FITC staining (Green). (b) Pearson coefficient analysis among different conditions showing the correlation between TILs infiltration with total number of TILs per chamber. Scale bars in a) represent 100 µm.

**
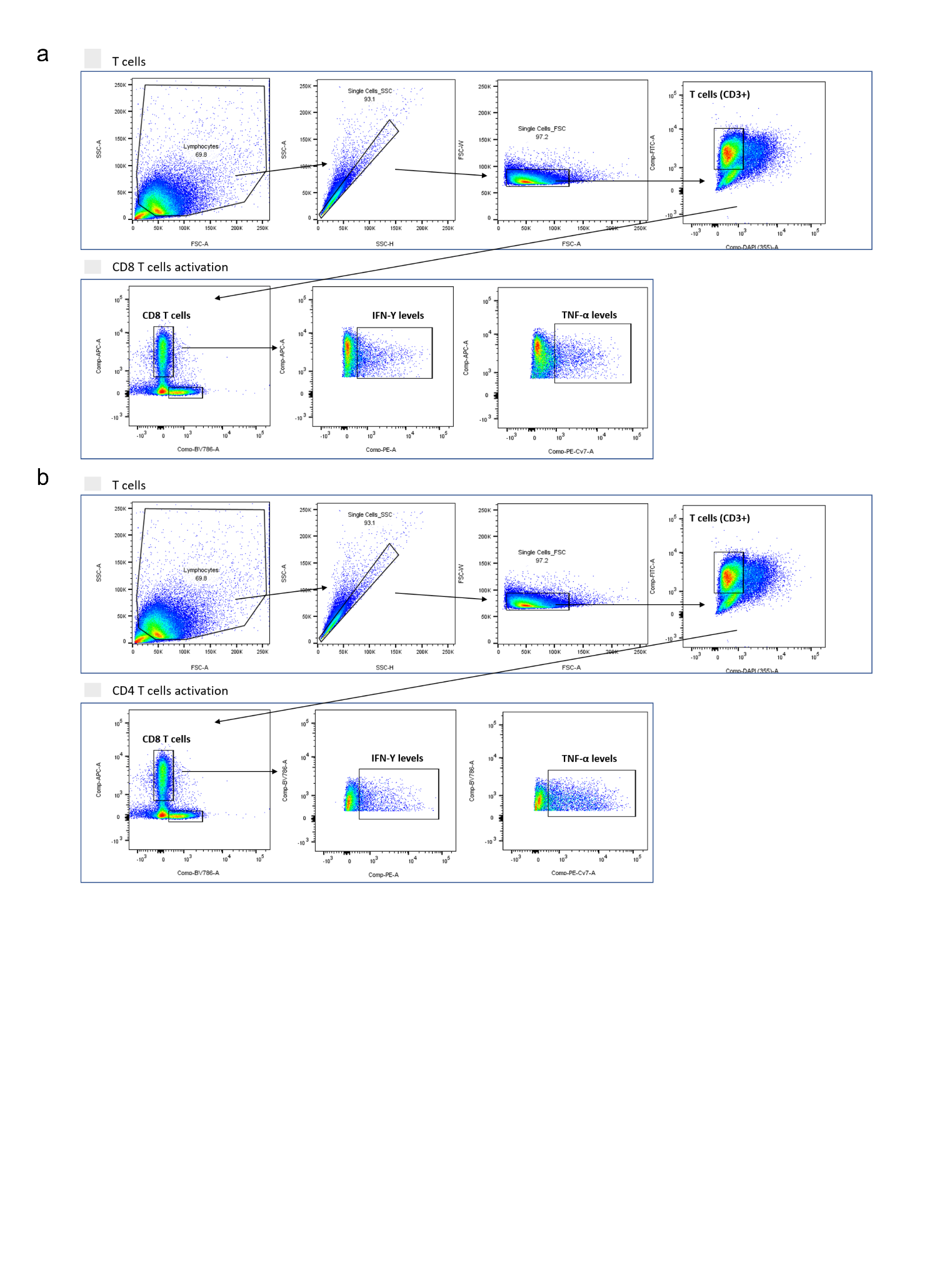
**

**Figure S4. Gating scheme for flow cytometric analysis of CD4T (a) and CD8T (b) cell reactivity from *in vitro* stimulated unfractionated human PBMCs.** (a) Doublets were excluded via SSC-A vs. SSC-H and FSC-A vs.FFC-H. Viable T-cells were identified in a CD3 vs. DAPI plot. CD8 T-cells were gated from viable T-cells and were identified in CD8 vs. CD4 plot. IFN-γ and TNF-α levels were identified from CD8-positive T-cells. (b) Doublets were excluded via SSC-A vs. SSC-H and FSC-A vs.FFC-H. Viable T-cells were identified in a CD3 vs. DAPI plot. CD4 T-cells were gated from viable T-cells and were identified in a CD8 vs. CD4 plot. IFN-γ and TNF-α levels were identified from CD4-positive cells.


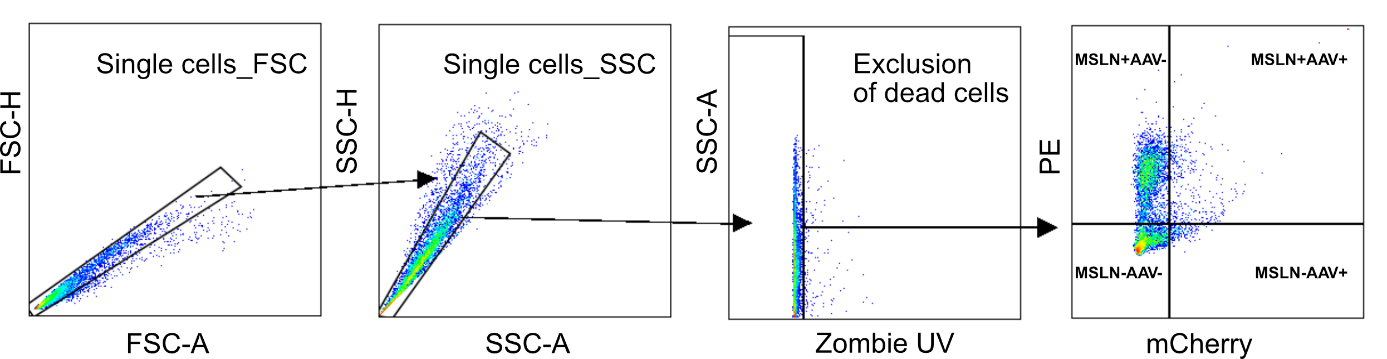


**Figure S5. Gating scheme for flow cytometric analysis of MSLN from transfected human PDOs.** Doublets were excluded via SSC-A vs. SSC-H and FSC-A vs.FFC-H. Viable tumor cells were identified as SSC-A vs. Zombie UV plot. MSLN-positive cells were identified in PE (MSLN) vs. mCherry (AAV) plot.


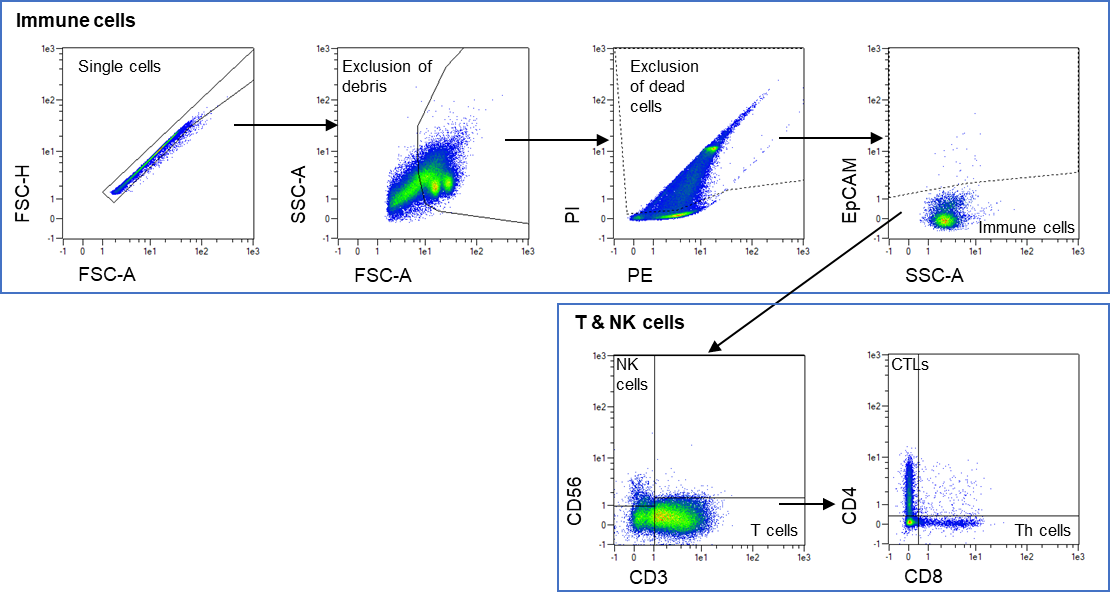


**Figure S6. Gating scheme for flow cytometric analysis of immune cell infiltration into human organoids in co-cultures.** Doublets were excluded as initial steps via FSC-A vs. FSC-H, followed by debris (based on low size and granularity; FSC-A vs. SSC-A) and exclusion of dead cells (dashed “not-gate” on PI-positive cells). Immune cells were identified as EpCAM-negative cells (“not-gate” in SSC-A vs. EpCAM). Among immune cells, NK cells and T-cells were identified (CD3 vs. CD56 plot), followed by identification of Th cells and CTLs in a CD4 vs. CD8 plot again based on viable T-cells.


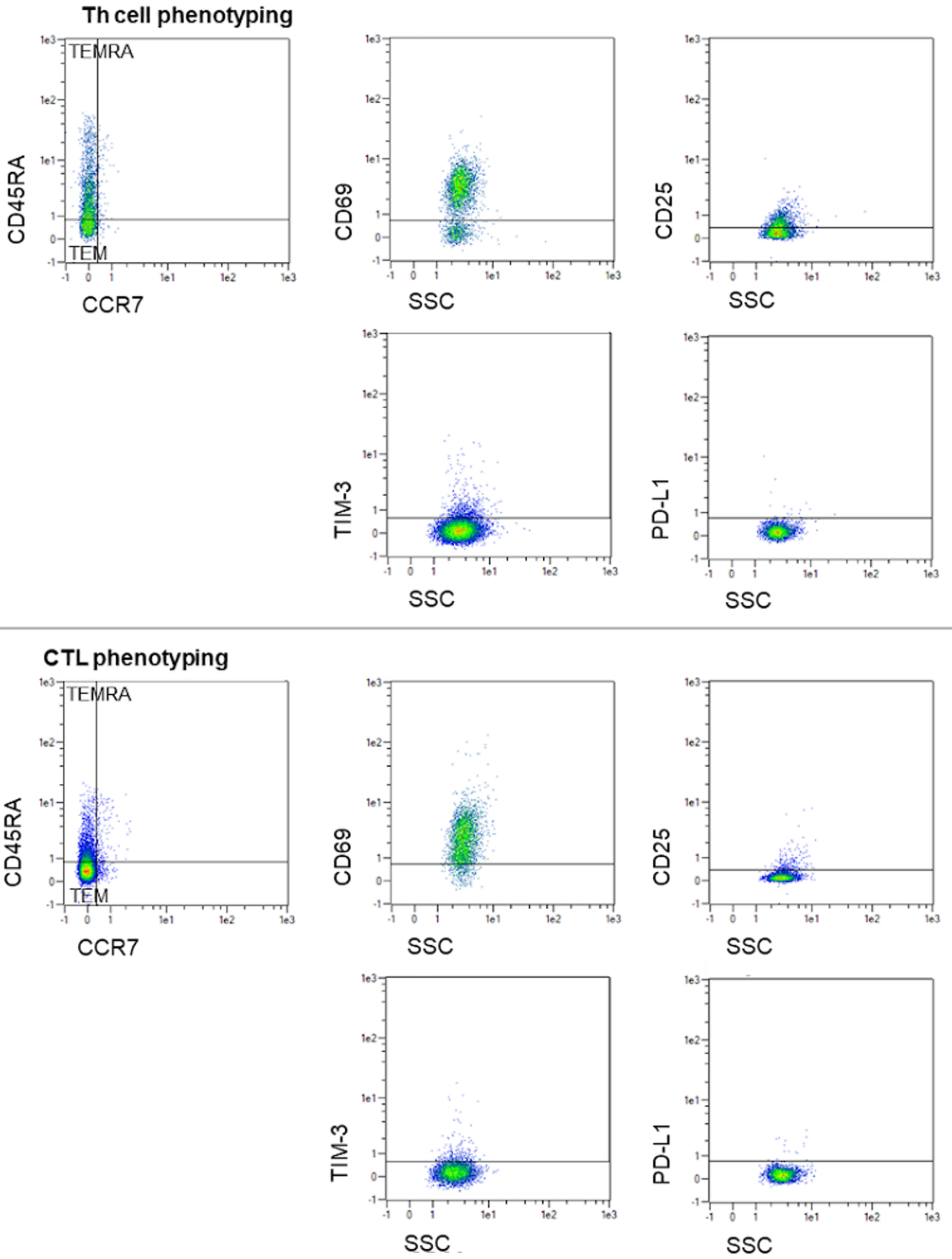


**Figure S7. Gating scheme for phenotyping of Th cells and CTLs from co-cultures with human organoids.** Th cells were characterized as shown in S2. By plotting CCR7 vs. CD45RA, naïve (CCR7+ CD45RA+), memory (CCR7+ CD45RA-), effector (CCR7- CD45RA-) and RA-expressing effector (CCR7- CD45RA+) T-cells were characterized. Activation was analyzed based on CD69 and CD25 expression. Exhaustion was assessed via PD-L1 and TIM-3 expression.

**
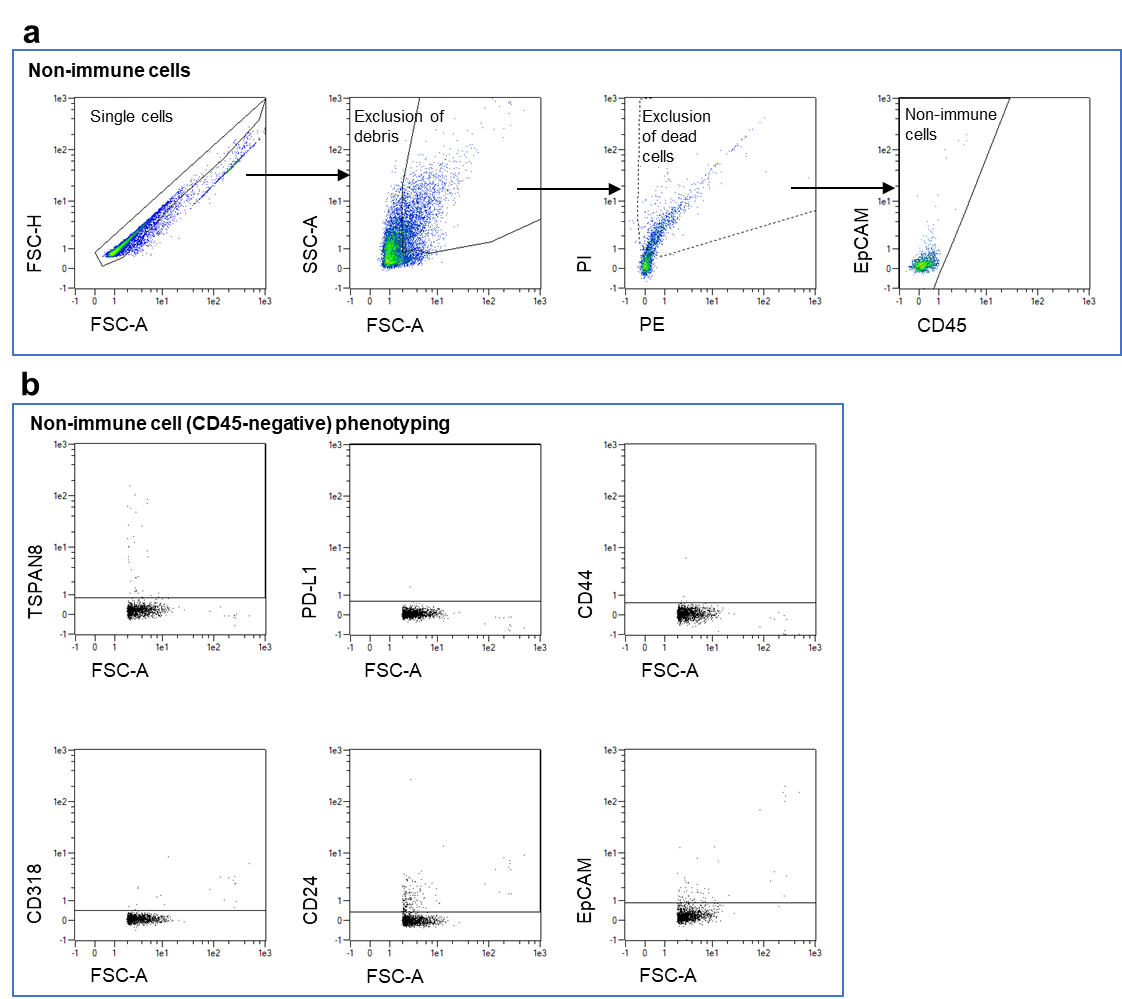
**

**Figure S8. Gating scheme for flow cytometric analysis of non-immune cells from human organoids co-cultures.** (a) Doublets were excluded, followed by debris- and immune cell- (FSC-A vs. SSC-A) and dead cell- exclusion (“non-gate” on PI-positive cells). Pancreatic epithelial (tumor) cells were identified as non-immune (CD45-) cells in a CD45 vs EpCAM plot. (b) Non-immune cells were analyzed for their expression of markers associated with pancreatic (tumor) cells.

**
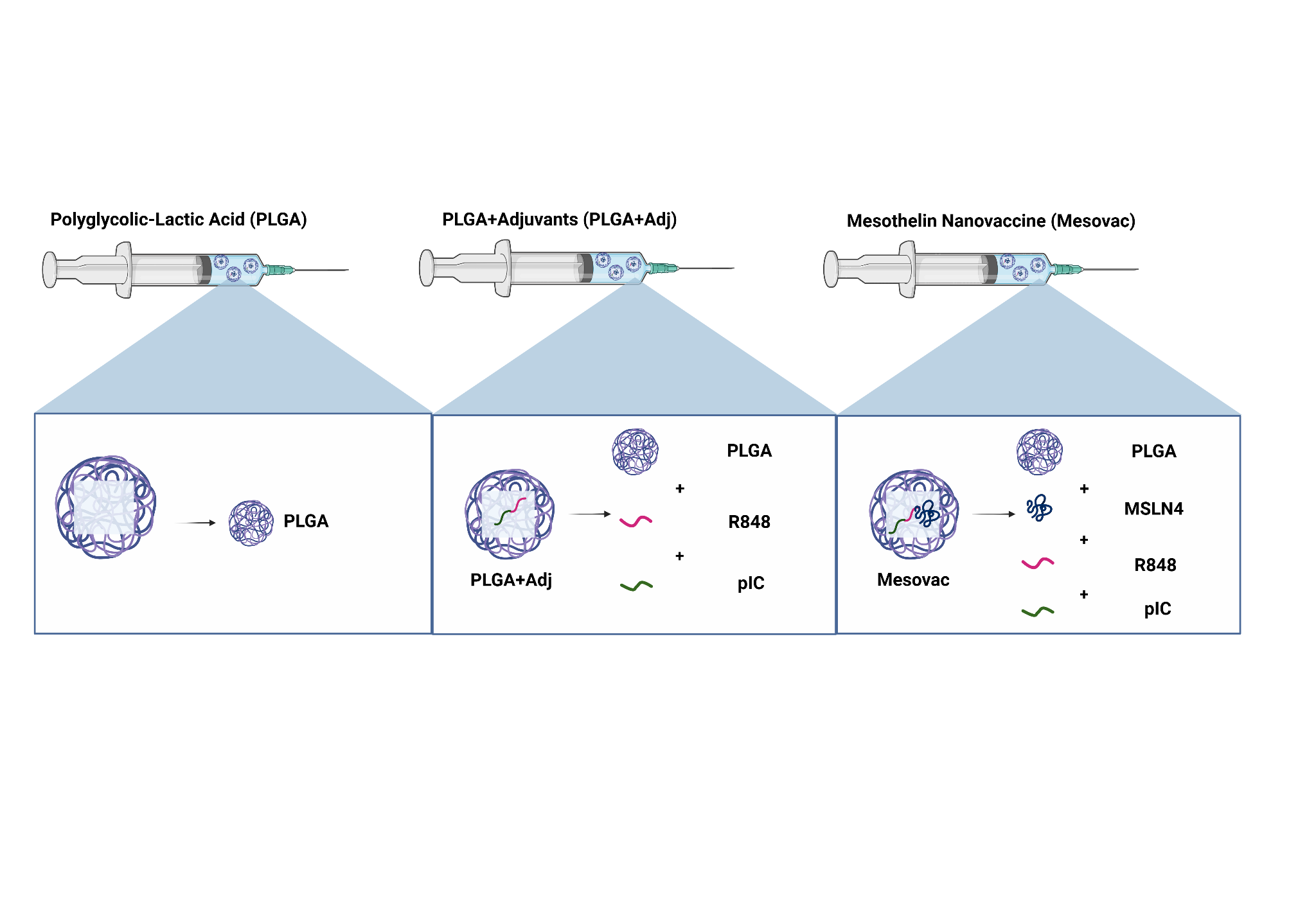
**

**Figure S9. Schematic illustration of the Mesothelin nanovaccine (Mesovac) formulation and corresponding controls**. Mesovac contains three main components: the MSLN4 peptide (Table 1), as well as Polyinosinic-polycytidylic acid (pIC) and Resiquimod (R848) as immune adjuvants encapsulated in PLGA. PLGA+Adj, containing only either pIC or R848 alone or both encapsulated in PLGA, and PLGA alone (PLGA) serve as control formulations for Mesovac. Created with BioRender.com.


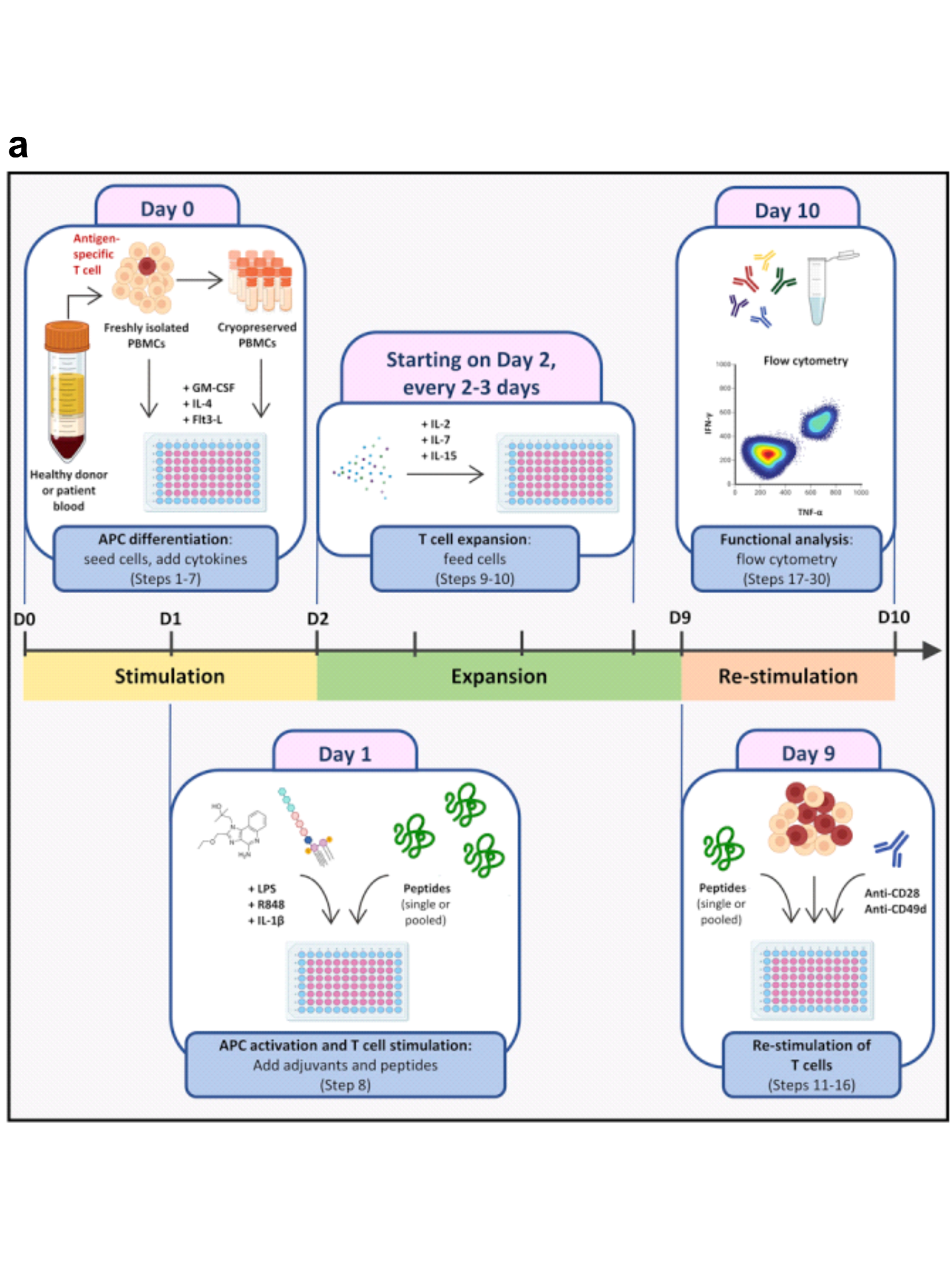


**Figure S10. Workflow for the stimulation of reactive T-cells from unfractionated PBMCs obtained from healthy donors**. This graphic was taken unchanged from Bozkus et al, STAR Protocols, Volume 2, Issue 3, 2021^[3]^. <https://creativecommons.org/licenses/by-nc-nd/4.0/>.

**
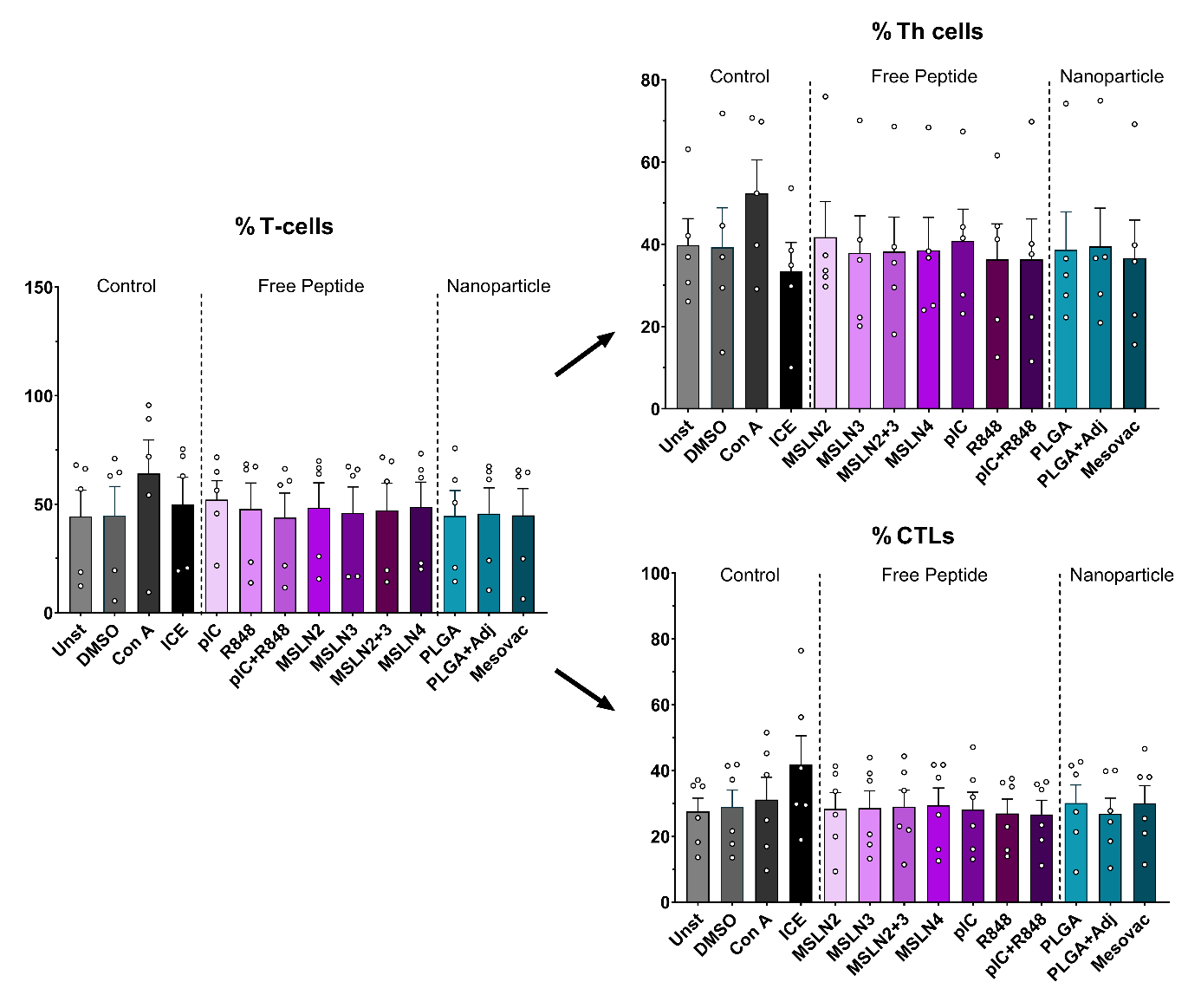
**

**Figure S11. No significant change was observed in the overall T-cell population following stimulation with the Mesovac peptide pool or the complete formulation.** Flow cytometry analysis of PBMCs stimulated either with each of the free peptides (MSLN2, MSLN3, MSLN2+3, and MSLN4) or the different nanoparticles (PLGA, PLGA+Adj, and Mesovac). T-cells (% viable CD3+ cells, left graph) were further categorized into Th cells (upper graphs) and CTLs (lower graphs).

References:

[1] S. S. Wilson, M. Mayo, T. Melim, H. Knight, L. Patnaude, X. Wu, L. Phillips, S. Westmoreland, R. Dunstan, E. Fiebiger, S. Terrillon, *Front Immunol* **2020**, *11*, 547102.

[2] L. Klemke, J. P. Blume, T. De Oliveira, R. Schulz-Heddergott, *Bio Protoc* **2022**, *12*, e4298.

[3] C. Cimen Bozkus, A. B. Blazquez, T. Enokida, N. Bhardwaj, *STAR Protoc* **2021**, *2*, 100758.
